# Macrophage-produced VEGFC is induced by efferocytosis to ameliorate cardiac injury and inflammation

**DOI:** 10.1172/JCI140685

**Published:** 2022-05-02

**Authors:** Kristofor E. Glinton, Wanshu Ma, Connor Lantz, Lubov S. Grigoryeva, Matthew DeBerge, Xiaolei Liu, Maria Febbraio, Mark Kahn, Guillermo Oliver, Edward B. Thorp

**Affiliations:** 1 Department of Pathology,; 2 Feinberg Cardiovascular and Renal Research Institute, and; 3 Department of Medicine, Feinberg School of Medicine, Northwestern University, Chicago, Illinois, USA.; 4 Department of Dentistry and Dental Hygiene, University of Alberta, Edmonton, Alberta, Canada.; 5 Department of Medicine, Perelman School of Medicine, University of Pennsylvania, Philadelphia, Pennsylvania, USA.; 6 Department of Pediatrics, Feinberg School of Medicine, Northwestern University, Chicago, Illinois, USA.; 7 The Heart Center at Ann & Robert H. Lurie Children’s Hospital of Chicago, Chicago, Illinois, USA.

**Keywords:** Inflammation, Vascular Biology, Innate immunity, Macrophages, Molecular pathology

## Abstract

Clearance of dying cells by efferocytosis is necessary for cardiac repair after myocardial infarction (MI). Recent reports have suggested a protective role for vascular endothelial growth factor C (VEGFC) during acute cardiac lymphangiogenesis after MI. Here, we report that defective efferocytosis by macrophages after experimental MI led to a reduction in cardiac lymphangiogenesis and *Vegfc* expression. Cell-intrinsic evidence for efferocytic induction of *Vegfc* was revealed after adding apoptotic cells to cultured primary macrophages, which subsequently triggered *Vegfc* transcription and VEGFC secretion. Similarly, cardiac macrophages elevated *Vegfc* expression levels after MI, and mice deficient for myeloid *Vegfc* exhibited impaired ventricular contractility, adverse tissue remodeling, and reduced lymphangiogenesis. These results were observed in mouse models of permanent coronary occlusion and clinically relevant ischemia and reperfusion. Interestingly, myeloid *Vegfc* deficiency also led to increases in acute infarct size, prior to the amplitude of the acute cardiac lymphangiogenesis response. RNA-Seq and cardiac flow cytometry revealed that myeloid *Vegfc* deficiency was also characterized by a defective inflammatory response, and macrophage-produced VEGFC was directly effective at suppressing proinflammatory macrophage activation. Taken together, our findings indicate that cardiac macrophages promote healing through the promotion of myocardial lymphangiogenesis and the suppression of inflammatory cytokines.

## Introduction

Heart failure after myocardial infarction (MI) remains a significant cause of morbidity and mortality (1, 2). Although pharmacological advances including beta blockers and angiotensin-converting enzyme (ACE) inhibitors (3) reduce mortality, the residual risk of post-MI heart failure remains high. Therefore, additional studies aimed at better characterizing the basic mechanisms of cardiac repair should facilitate the development of novel and complementary approaches to preserve heart function.

Acute MI mobilizes the accumulation of cardiac macrophages (4). These innate phagocytes are required to promote myocardial healing (5). One mechanism by which myocardial macrophages coordinate cardiac repair is through the process of efferocytosis, or the clearance of dying cells. Supporting this argument, defects in efferocytosis have been found to lead to accelerated heart failure (6, 7). Furthermore, efferocytosis is the first step in a process of antigen trafficking to lymph nodes (LNs). Trafficking of cardiac antigens by phagocytes has the capacity to activate either Tregs or effector T cells, thus calibrating the myocardial inflammatory response (8).

In the heart, important new data have recently emerged about the functional roles of cardiac lymphatics. For example, MI induces expression of the pro-lymphangiogenic factor vascular endothelial growth factor C (VEGFC) that in turn triggers cardiac lymphangiogenesis, leading to improved cardiac function (9). Interestingly, phagocytes, and in particular CD11b^+^ macrophages, have been reported to secrete VEGFC and promote lymphangiogenesis during inflammation (10, 11). Furthermore, the cardiac lymphatic vasculature has been linked to the resolution of inflammation following MI (12). Here, we describe studies linking efferocytosis to the induction of *Vegfc* expression as well as a contributing role for myeloid *Vegfc* in cardiac repair and the regulation of cytokine production.

## Results

### CD36 regulates Vegfc expression after MI.

**Figure 1:**
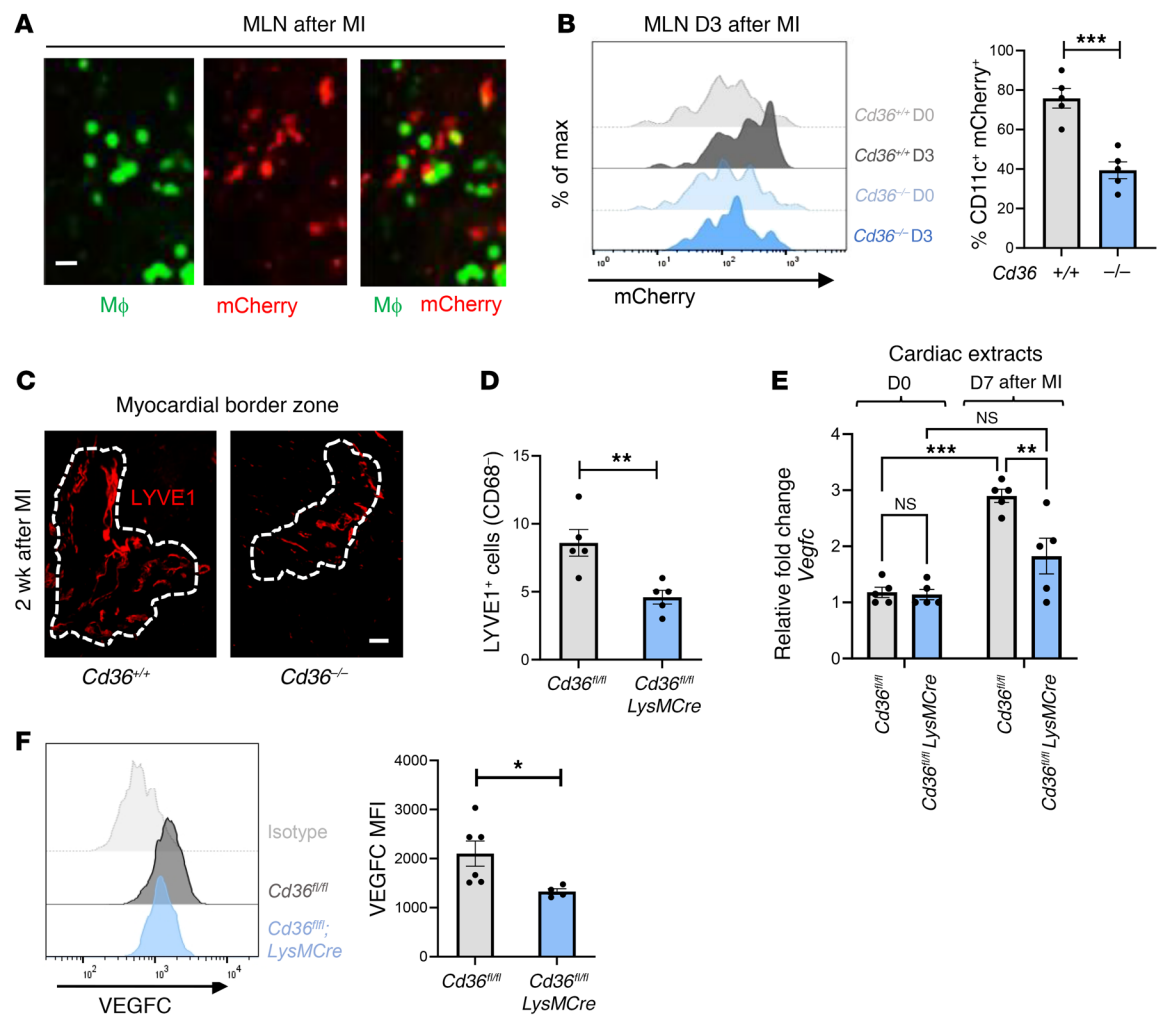
Bone marrow–derived *Cd36* is required for both the accumulation of cardiac antigens in MLNs and increased tubular LYVE1 staining after MI. (**A**) Imaging of murine MLN cross-sections 3 days after ligation of the LAD artery. Macrophages (MΦ) from *LysMCre-EGFP* mice show EGFP signal, and cardiomyocyte debris from *Myh6-mCherry*–transgenic mice show red signal. Scale bar: 40 μm. (**B**) Chimeric *Myh6-mCherry* mice, deficient for bone marrow–derived *Cd36*, were subjected to coronary artery occlusion. Three days after MI (D3), MLNs were harvested, and flow cytometric analysis of Ly6g^–^CD11b^+^CD11c^+^ cells was performed. *n =* 5 per group. max, maximum. ***P < 0.0005, by 2-tailed, unpaired *t* test. (**C**) C57BL/6 mice were subjected to MI via coronary occlusion of the LAD artery. Representative immunofluorescence images were taken from 2 weeks after MI. Data display tubular LYVE1^+^ staining of the myocardial infarct border zone in *Cd36^–/–^* mice versus *Cd36^+/+^* mice. Scale bar: 125 μm. (**D**) Border zone quantification of LYVE1^+^CD68^–^ nuclei in *Cd36^fl/fl^* versus *Cd36^fl/fl^* *LysMCre* mice after MI. *n* = 5 per group. **P < 0.007, by 2-tailed, unpaired *t* test. (**E**) qPCR analysis of myocardial *Vegfc* in *Cd36-*deficient mice after MI. *n =* 5 per group. **P < 0.005 and ***P < 0.0001, by 2-way ANOVA followed by Tukey’s test. (**F**) Expression of VEGFC in cardiac macrophages with myeloid-specific deletion of *Cd36* compared with controls 7 days after MI. *n =* 4–6 mice per group pooled from 2 independent experiments. *P < 0.05, by 2-tailed, unpaired *t* test.

Following cardiac injury, we and others have reported that phagocytic clearance of dying cells by macrophages (i.e., efferocytosis) is a necessary initial step for cardiac repair (6, 7). Efferocytosis also leads to the transport of cardiac antigen to draining LNs (13), where it may be cross-presented to resident T cells (14). To track the fate of phagocytosed cardiac antigen, we harvested mediastinal lymph nodes (MLNs) and spleens prior to and after experimental MI. As previously demonstrated (15), we identified cardiac-derived (Myh6-mCherry) antigens at steady state in MLNs (Supplemental Figure 1A; supplemental material available online with this article; https://doi.org/10.1172/JCI140685DS1). As shown in Supplemental Figure 1A, this mCherry signal was greatly enhanced in MLNs following ligation of the left anterior descending (LAD) artery after 3 days, however, levels in the spleen remained negligible. mCherry signal also colocalized with MLN *LysMCre-EGFP^+^* phagocytes (Figure 1A and ref. 16). Here, we sought to decipher whether *Cd36* was necessary for the transport of cardiac antigen to the MLNs after MI. Consistent with a phagocytic origin, flow cytometric analysis of MLNs revealed that cardiac mCherry antigen within draining LNs was reduced in mice deficient for *Cd36* (Figure 1B).

We next examined the origin of the MLN cardiac antigen and performed imaging studies of the myocardium after MI. In line with prior reports indicating that cardiac damage induces a lymphangiogenesis response in association with production of the lymphangiogenic factor *Vegfc* (9), we also found increased expression of typical lymphatic vascular markers such as lymphatic vessel endothelial hyaluronan receptor 1 (LYVE1) (Supplemental Figure 1B) and *Prox1Td-tomato* (Supplemental Figure 1C) in myocardial lymphatic networks following MI. We further confirmed that the LYVE1-expressing lymphatics did not coexpress the pan-macrophage marker CD68 (Supplemental Figure 1D), given that LYVE1 may also be produced by cardiac macrophages (17). We then revisited the *Cd36-*deficient mice and found that myocardial LYVE1 staining was significantly reduced in these mice after MI (Figure 1, C and D). These initial data suggested that reduced cardiac antigen accumulation in MLNs of *Cd36-*deficient mice (Figure 1B) might not be a direct consequence of reduced phagocytosis, as we had initially predicted. Consistent with an alternative mechanism, *Cd36^fl/fl^* *LysMCre* mice exhibited a reduction in myocardial *Vegfc* expression relative to control mice after MI (Figure 1E). Finally, cardiac macrophages from *Cd36-*deficient animals exhibited reduced VEGFC expression as measured by flow cytometry (Figure 1F). Altogether, these data suggest that myeloid CD36 regulates *Vegfc* expression after MI.

### Efferocytosis triggers macrophage production of VEGFC in a CD36-dependent manner.

**Figure 2:**
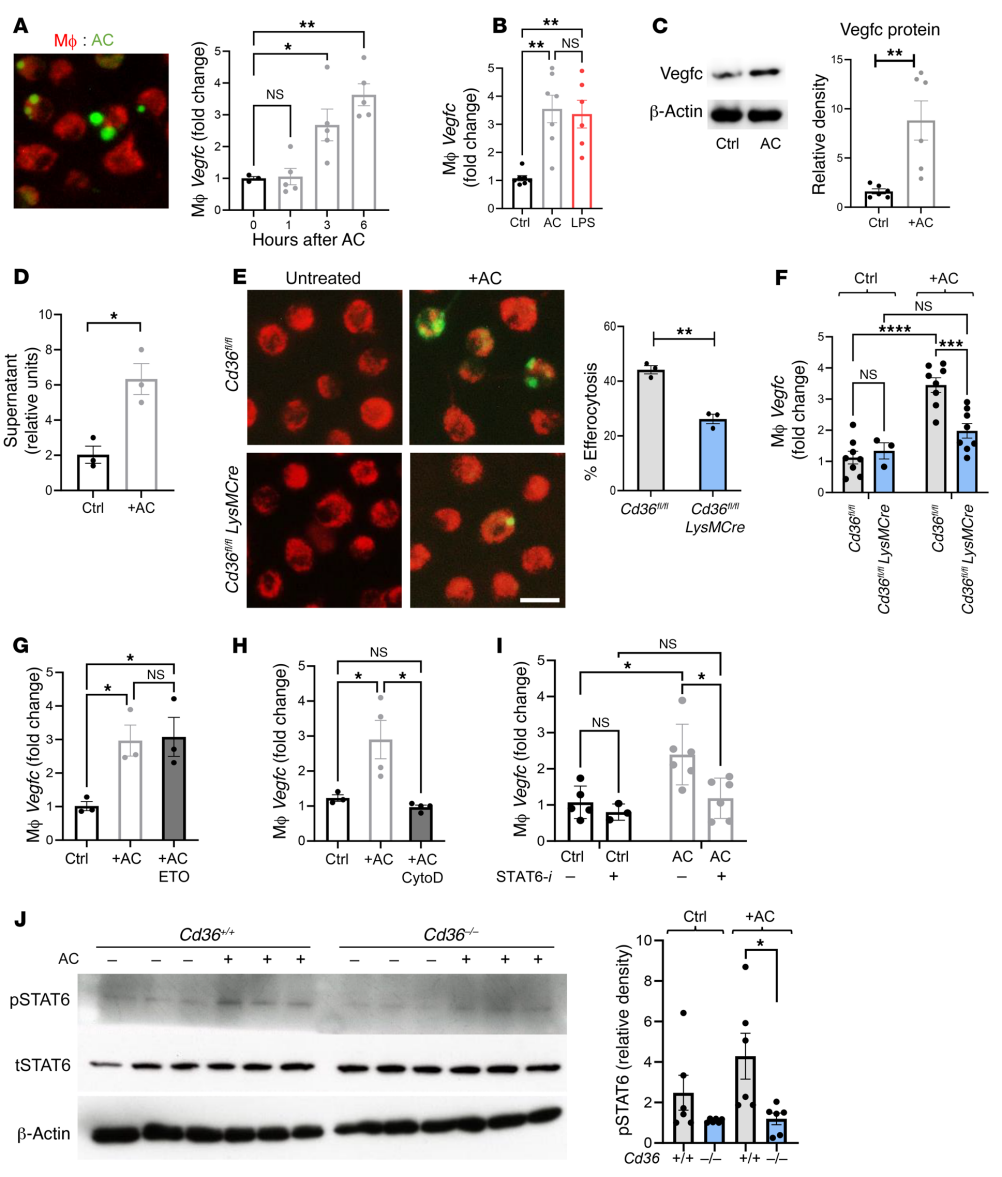
*Vegfc* is induced in macrophages during efferocytosis. (**A**) Photomicrograph depicts primary BMDMs (red) cocultivated with fluorescently labeled (green) ACs. Original magnification, ×40. Bar graph shows the quantification of gene expression at the indicated time points after AC cocultivation. *n =* 3–5. *P < 0.03 and **P < 0.003, by 1-way ANOVA with Tukey’s test. (**B**) Macrophage gene expression after treatment with either ACs or LPS (100 ng/mL). *n =* 6 per group. **P < 0.006. (**C**) Representative protein immunoblots of VEGFC, 6 hours after efferocytosis and densitometric analysis. *n =* 6 pooled from 2 independent experiments. **P < 0.005. (**D**) VEGFC ELISA of macrophage supernatant 9 hours after treatment with ACs versus control. *n =* 6. *P < 0.01. (**E**) BMDMs from *Cd36^+/+^* and *Cd36^–/–^* animals were cocultured for 3 hours with ACs. After sequential washes, cells were imaged on an Olympus fluorescence microscope, and the percentage of efferocytosis was calculated from 10 random fields per replicate. Scale bar: 20 μm. Data are representative of 2 independent experiments with *n =* 3 wells per group. **P < 0.01. (**F**) BMDMs from *Cd36^+/+^* and *Cd3^–/–^* animals was assessed for gene expression before and after treatment with ACs. *n =* 3–8 per group. ***P < 0.0005 and ****P < 0.0001. (**G**) Macrophage *Vegfc* gene expression after treatment with either ACs or with ACs plus etomoxir (ETO). *n =* 3–4 wells per group. *P < 0.01. (**H**) Quantification of gene expression in macrophages treated with ACs versus ACs plus cytochalasin D (CytoD). *n =* 3 per group. *P < 0.02. (**I**) Inhibition of STAT6 with AS1517499 blocked efferocytic *Vegfc* induction. *P < 0.01. (**J**) To assess STAT6 phosphorylation, macrophages were cultured as above, and lysates were prepared in RIPA buffer and then assessed by Western blotting. *n =* 6 pooled samples from 2 independent experiments. *P < 0.05. Data are presented as the mean ± SEM. Statistical significance was determined by 2-way ANOVA followed by Tukey’s test (**B** and **F**–**J**) and 2-tailed, unpaired *t* test (**C**–**E**). Ctrl, control.

Macrophage-like cells have been shown to have the capacity to produce VEGFC (10). However, in the setting of MI, the source of cardiac VEGFC and the trigger of lymphangiogenesis remain unclear. Because of the role of CD36 in apoptotic cell (AC) clearance, our observations in *Cd36*-deficient mice led us to question whether a mechanistic link exists between efferocytosis and *Vegfc* expression. To determine whether, similar to tissue injury, efferocytosis could trigger the induction of *Vegfc*, we cocultivated primary macrophages isolated from the bone marrow of *C57BL/6J* mice with apoptotic Jurkat cells and measured *Vegfc* levels by quantitative PCR (qPCR). Interestingly, we found that *Vegfc* levels were acutely elevated as a function of time after efferocytosis (Figure 2A). LPS has been reported to induce *Vegfc* in macrophages (18). We found that the increase in *Vegfc* levels after efferocytosis was similar to that seen in macrophages exposed to LPS (Figure 2B). Furthermore, we also observed an increase in VEGFC expression at the protein level (Figure 2C) and detected this protein in cell supernatants after performing an ELISA (Figure 2D). Supporting our in vivo data (Figure 1), *Cd36* was also required for optimal efferocytosis and an increase in *Vegfc* levels (Figure 2, E and F). Since *Cd36* also acts as a fatty acid transporter, we sought to determine whether *Vegfc* expression is affected by fatty acid oxidation (FAO). We found that the use of etomoxir, which can inhibit FAO (19), had no effect on *Vegfc* expression during efferocytosis (Figure 2G). Moreover, inhibition of AC engulfment by pretreatment of macrophages with the cytoskeleton inhibitor cytochalasin D, which prevents phagocytic uptake (20), completely abrogated AC-triggered induction of *Vegfc* (Figure 2H). To explore how efferocytosis might signal to activate *Vegfc*, we leveraged a separately conducted screen, in which we examined open chromatin marks during efferocytosis by ChIP using H3K27Ac antibodies (Supplemental Figure 2, A and B). These data were consistent with an activated *Vegfc* locus during efferocytosis (Supplemental Figure 2C). Also, DNA footprint analysis of candidate transcription factor binding sites in this area suggested a role during this process for the transcription factor *Stat6* (Supplemental Figure 2D). Indeed, inhibition of STAT6 impaired efferocytic *Vegfc* induction (Figure 2I). We next tested whether CD36 enhances efferocytosis-driven STAT6 signaling and observed diminished levels of phosphorylated STAT6 (p-STAT6) in *Cd36*-deficient macrophages compared with levels in control macrophages (Figure 2J). Thus, CD36-dependent efferocytosis triggers STAT6 activation, leading to macrophage production of VEGFC.

### Myeloid-derived VEGFC regulates post-MI lymphangiogenesis and cardiac function.

**Figure 3:**
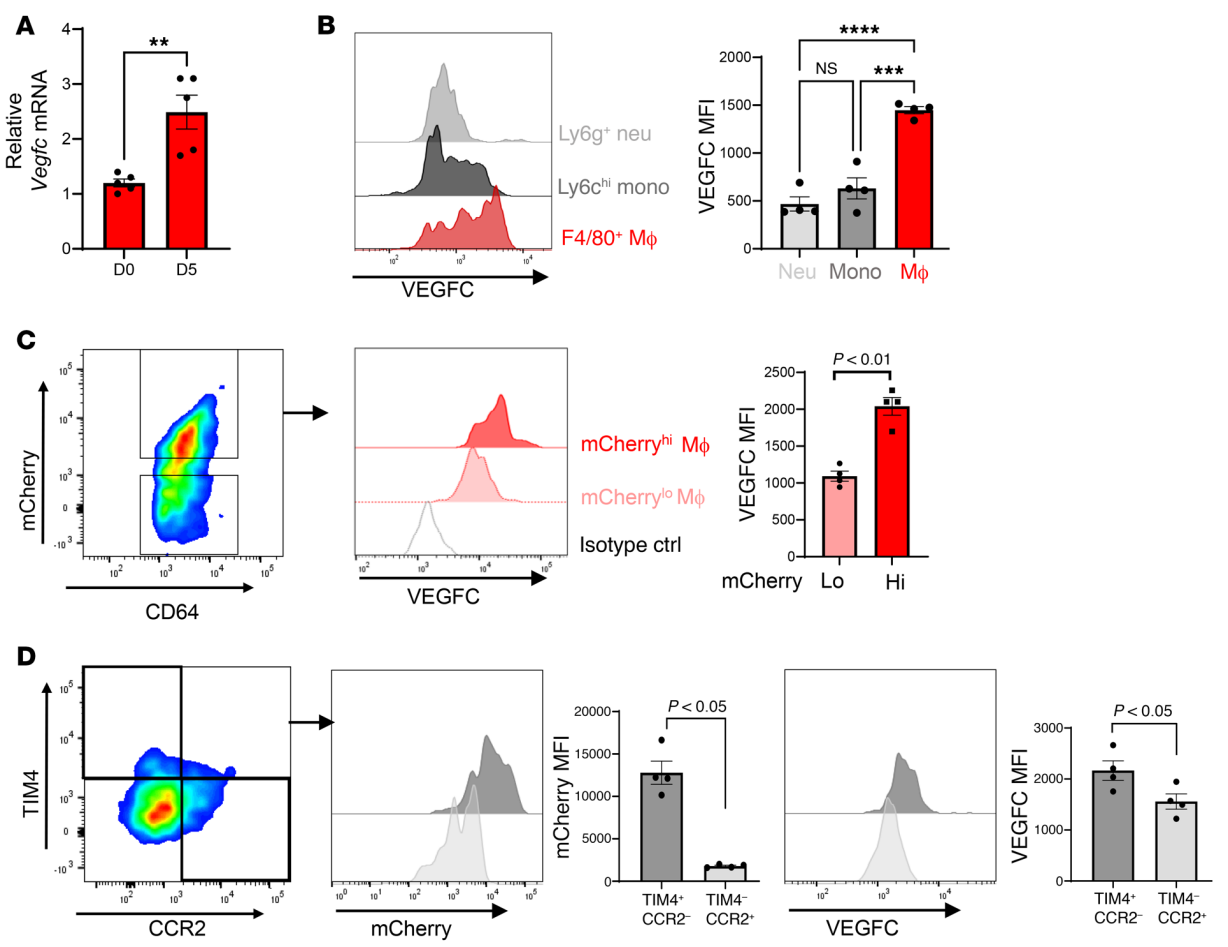
Selective expression of *Vegfc* in cardiac macrophages after MI. (**A**) Sorted cardiac macrophages 5 days after MI were assessed for *Vegfc* mRNA expression compared with non-MI mice. *n =* 5/group. **P < 0.005, by unpaired *t* test. (**B**) Histogram of VEGFC expression in the indicated cell types by quantitative flow cytometry using cardiac extracts, 7 days after MI. Neutrophils (Neu) were defined as CD11b^+^Ly6g^+^; monocytes (Mono) were CD11b^+^Ly6g^–^Ly6c^hi^F4/80^–^; macrophages (Mac) were defined as CD11b^+^Ly6g^–^Ly6c^lo^F4/80^+^CD64^+^. *n =* 4 per group. ***P < 0.0002 and ****P < 0.0001, by 2-way ANOVA followed by Tukey’s test. (**C**) mCherry mice were subjected to coronary ligation to track the uptake of cardiac antigens. Cardiac macrophages with higher levels of mCherry signal also expressed higher levels of VEGFC. *n =* 4 per group. *P <* 0.01, by 2-tailed, unpaired *t* test. (**D**) Cardiac macrophages were further classified by TIM4 (resident) or CCR2 (recruited) expression. TIM4^+^ resident macrophages had a higher frequency of mCherry uptake and expressed higher levels of VEGFC. *n =* 4 per group. Data were pooled from 2–3 independent experiments. *P <* 0.05, by 2-tailed, unpaired *t* test. Data are presented as the mean ± SEM.

Next, we sought to validate our in vitro findings in mice. As displayed in Figure 3A, sorted cardiac macrophages showed significantly increased *Vegfc* levels 5 days after MI. We also compared *Vegfc* expression in sorted cardiac neutrophils, monocytes, and macrophages and discovered a selective macrophage *Vegfc* expression signature 7 days after MI (Figure 3B). Using *Myh6-mCherry* mice, we stratified separate macrophage subpopulations by the levels of mCherry signal as a readout of efferocytic uptake. We found that mCherry^hi^ macrophages had higher *Vegfc* signal than did mCherry^lo^ populations (Figure 3C). We further parsed the cardiac macrophages using reported markers of resident (TIM4^+^CCR2^–^) or recruited (TIM4^–^CCR2^+^) macrophages. Consistent with our previous findings (21), and in agreement with greater *Vegfc* production, CCR2^–^ macrophages exhibited enhanced efferocytic activity compared with recruited CCR2^+^ macrophages (Figure 3D). Supporting the idea of a potential crosstalk with cardiac lymphatics, CD68^+^ macrophages were also found proximal to cardiac LYVE1^+^ lymphatics in the heart (Supplemental Figure 3).

**Figure 4:**
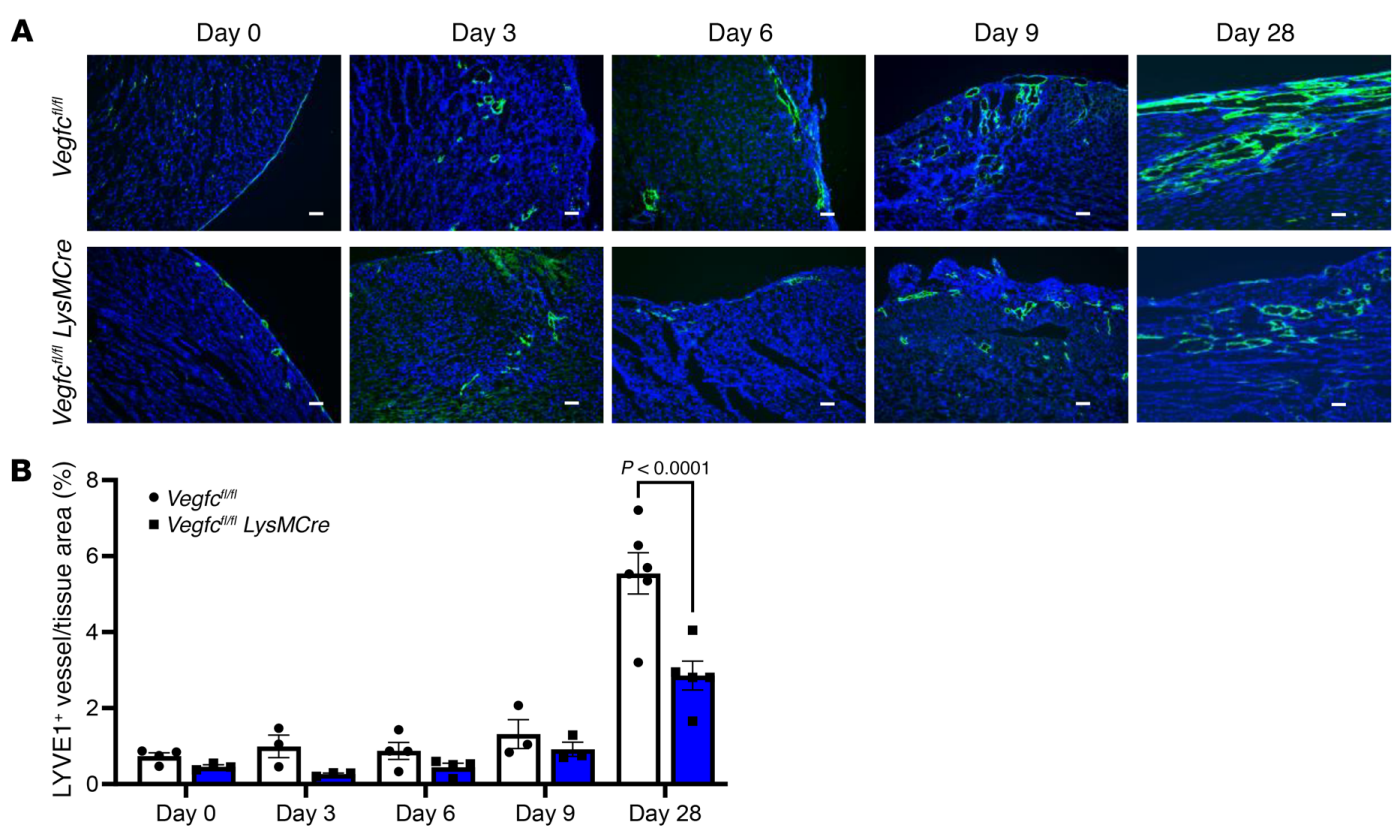
Myeloid deletion of *Vegfc* reduces lymphatic vessel density after MI. (**A**) Representative photomicrographs showing LYVE1 staining of coronary lymphatic vessels from *Vegfc^fl/fl^* *LysMcre* mice and *Vegfc^fl/fl^* littermates after permanent ligation of the LAD artery at the indicated time points. Scale bars: 50 μm. (**B**) Quantification of LYVE1 staining in myocardial ischemic AAR. *P <* 0.0001, by 2-way ANOVA followed by Tukey’s test. Data are presented as the mean ± SEM.

**Figure 5:**
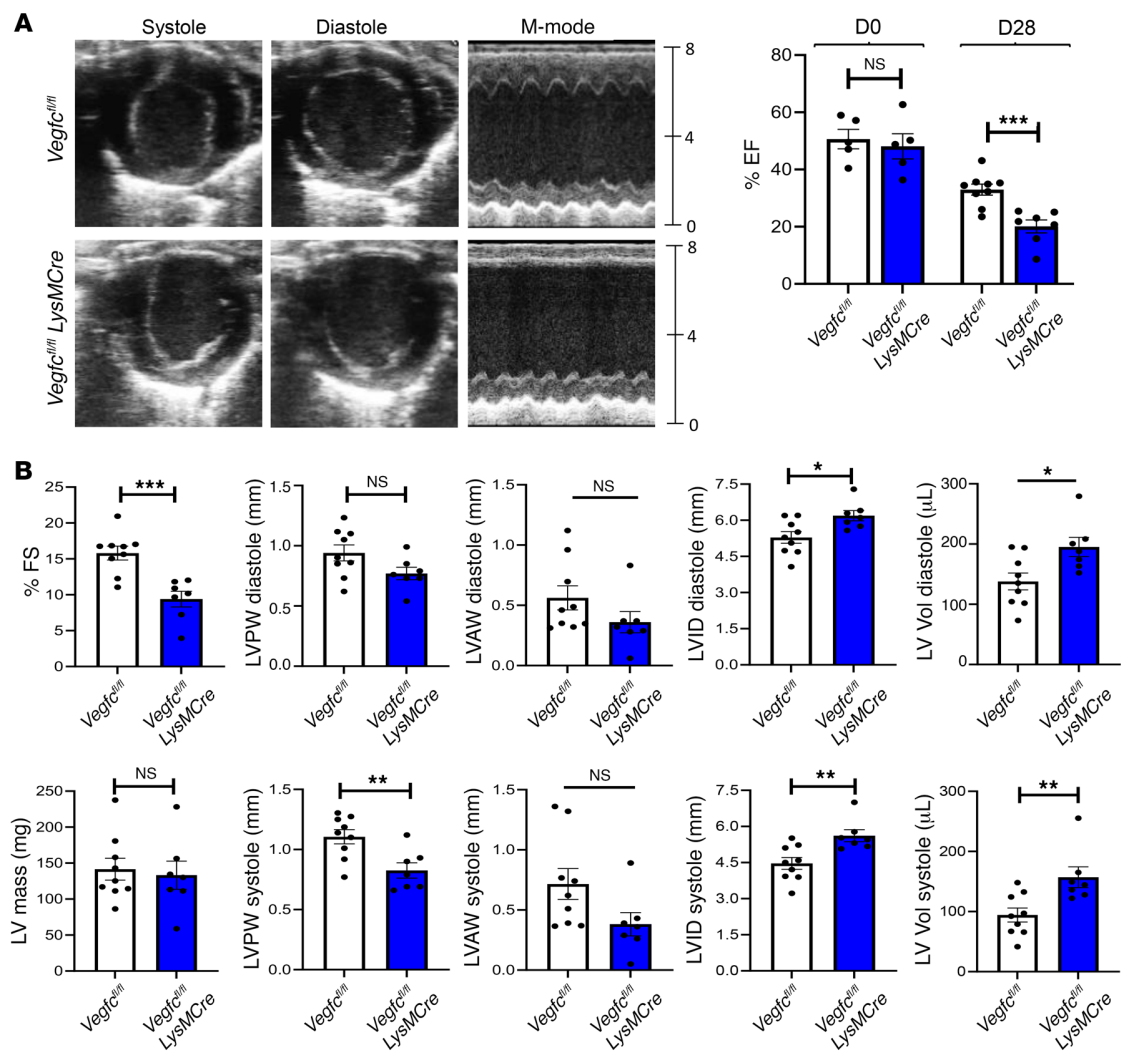
Myeloid *Vegfc* deficiency leads to impaired cardiac function after MI. (**A**) *Vegfc^fl/fl^* *LysMCre* mice along with littermate *Vegfc^fl/fl^* controls were subjected to permanent ligation of the LAD artery. Representative M-mode still frames used for analysis showed a significant reduction in ventricular wall thickness and contraction in *Vegfc*-deficient animals. Parasternal short-axis M-mode measurements were collected prior to surgery (day 0) and again on day 28 after the ligation procedure. Using EF measurements as an indicator of cardiac function, no inherent differences were observed prior to injury, however, after 28 days, the *Vegfc*-deficient animals showed a significant reduction in EF. *n =* 9 control *Vegfc^fl/fl^* controls; *n* = 7 *Vegfc^fl/fl^ LysMCre* mice. ***P < 0.005, by 2-tailed, unpaired *t* test. (**B**) Additional indices measured by echocardiography show significantly worsened indicators of systolic function including ventricular wall thickness, internal diameter, and volume. *P < 0.05, **P < 0.01, and ***P < 0.005, by 2-tailed, unpaired *t* test. FS, fractional shortening, LV Vol, left ventricular volume; LVID, left ventricular internal diameter; LVAW, left ventricular anterior wall thickness; LVPW, left ventricular posterior wall thickness.

**Figure 6:**
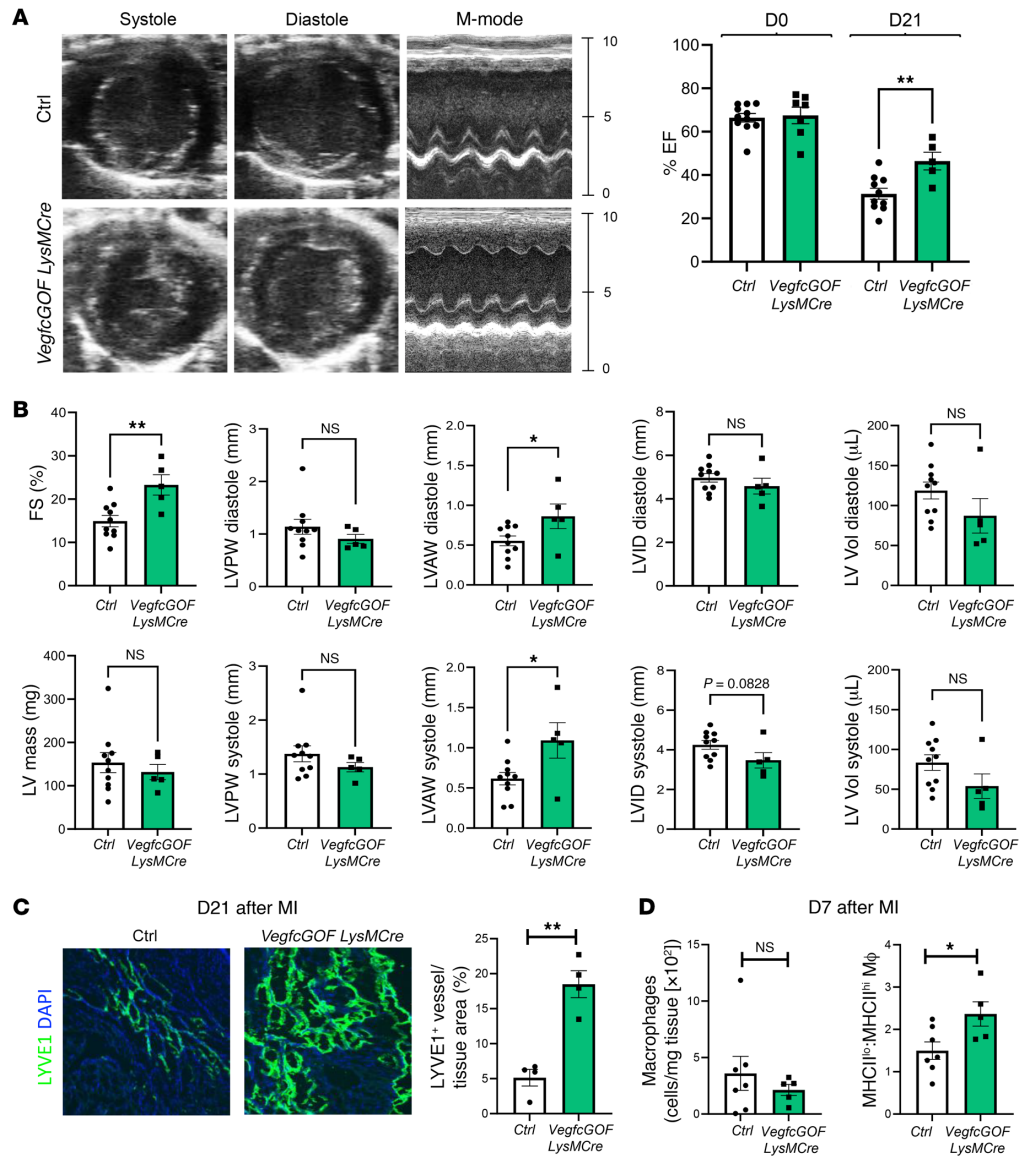
Myeloid *Vegfc* overexpression leads to improved cardiac function and increased lymphangiogenesis after MI. (**A**) *VegfcGOF* *LysMCre* mice and littermate controls were subjected to permanent ligation of the LAD artery. Parasternal short-axis M-mode measurements were collected prior to surgery (day 0) and again on day 21 after the ligation procedure to obtain EF measurements as an indicator of cardiac function. (**B**) Additional indices measured by echocardiography showed significantly improved indicators of systolic function including ventricular wall thickness, internal diameter, and volume. *n =* 9 control mice and *n* = 5 *VegfcGOF* *LysMCre* mice. (**C**) Representative photomicrographs and quantification of imaging from cardiac sections that were immunostained for LYVE1 in *VegfcGOF* *LysMCre* mice versus control, after MI. *n =* 4 per group. Original magnification, ×10. (**D**) Cardiac macrophages were assessed by flow cytometric analysis 7 days after MI. No significant differences in the absolute number of macrophages were observed. However, *VegfcGOF* *LysMCre* mice maintained a higher frequency of reparative MHCII^lo^ macrophages. *P < 0.05 and **P < 0.01, by 2-tailed, unpaired *t* test (**A**–**D**).

Because *Cd36* can affect multiple physiological functions, we sought to directly examine whether myeloid-derived *Vegfc* regulates the myocardial response to ischemia. We therefore compared *Vegfc^fl/fl^* *LysMCre* mice with *Vegfc^fl/fl^* controls after coronary artery ligation. As indicated in Figure 4, myeloid *Vegfc* deficiency led to reduced expression levels of cardiac LYVE1^+^ lymphatics, most notably within the ischemic area at risk (AAR). Importantly, we found that cardiac function was also affected, as the left ventricular ejection fraction (EF) was significantly reduced by myeloid *Vegfc* deficiency, as measured by M-mode echocardiography (Figure 5A). Additional echocardiographic parameters of ventricular remodeling were exacerbated in myeloid *Vegfc*–deficient mice, which, notably, had pronounced left ventricular dilation (Figure 5B and Supplemental Figure 4A). Consistent with a cardioprotective role of macrophage-produced *Vegfc* after MI, forced myeloid gain of function (GOF) of *Vegfc* using a *VegfcGOF* *LysMCre* mouse strain, led to an improvement in the post-MI cardiac EF (Figure 6, A and B). These myeloid *VegfcGOF* *LysMCre* mice also exhibited evidence of increased cardiac lymphatics in infarcted tissue (Figure 6C) and a higher frequency of cardiac macrophages with lower MHC class II (MHCII) expression (Figure 6D). To assess the potential clinical significance, we evaluated similar parameters after ischemia-reperfusion (I/R) and found that LYVE1 staining in myocardial lymphatics was also elevated in the AAR after I/R (Supplemental Figure 4). Echocardiography revealed that macrophage *Vegfc* was required for improved cardiac function after reperfusion of the ischemic hearts (Supplemental Figure 5).

### Myeloid-derived Vegfc is required for a post-MI immune response.

**Figure 7:**
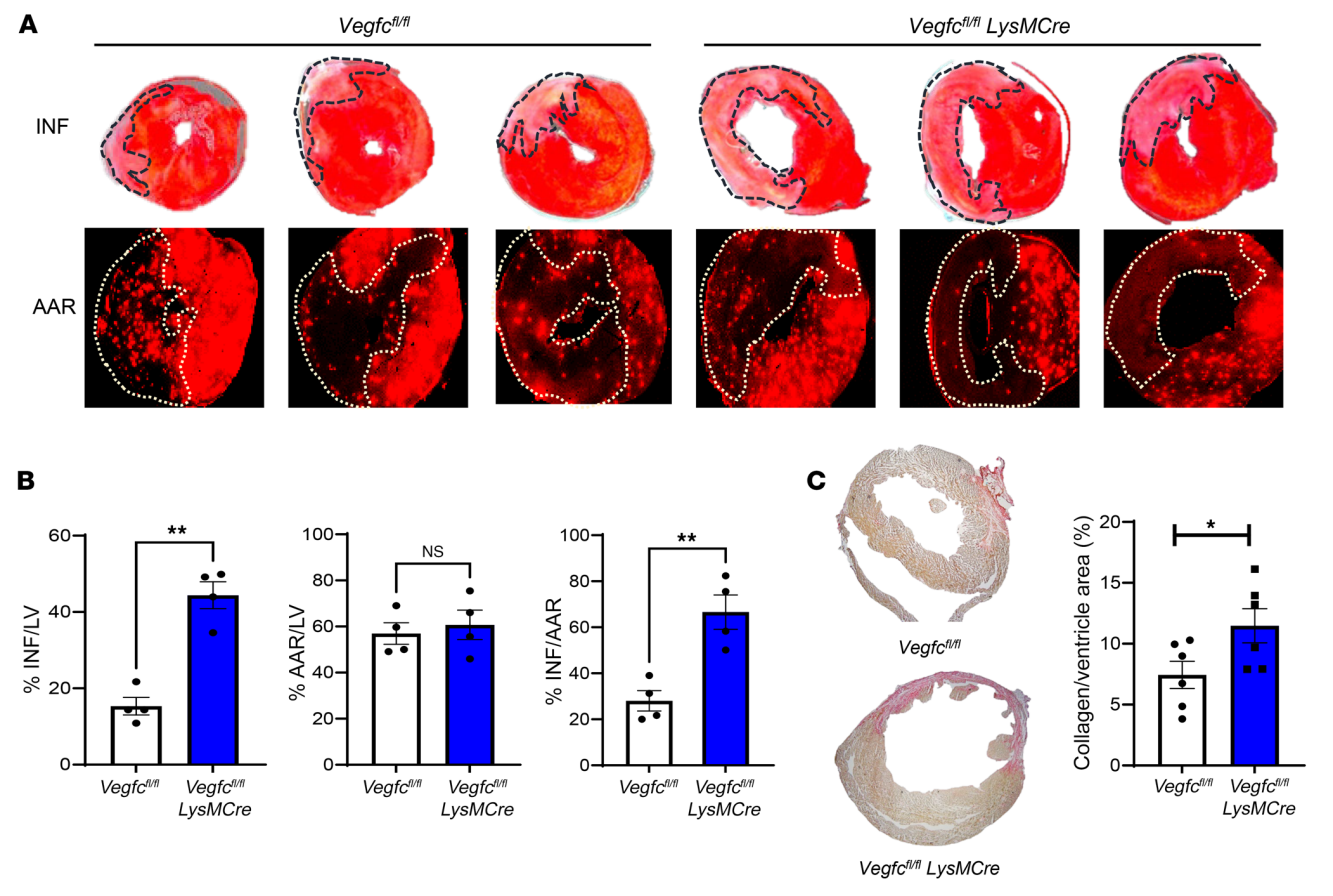
Myeloid-derived *Vegfc* ameliorates scarring and infarct size after MI. Mice of the indicated genotypes were subjected to experimental MI after ligation of the LAD artery. (**A**) The AAR was determined by intramyocardial circulation of fluorescent microbeads, and the infarct (INF) size was determined by TTC staining 7 days after the MI. (**B**) Quantification of the AAR and infarct size. *n =* 4 per group. **P < 0.0018, by 2-tailed, unpaired *t* test. (**C**) Representative Picrosirius red staining and quantification of fibrosis in cardiac sections on day 28 after the ligation procedure. *n =* 6 per group. *P < 0.05, by 2-tailed, unpaired *t* test.

**Figure 8:**
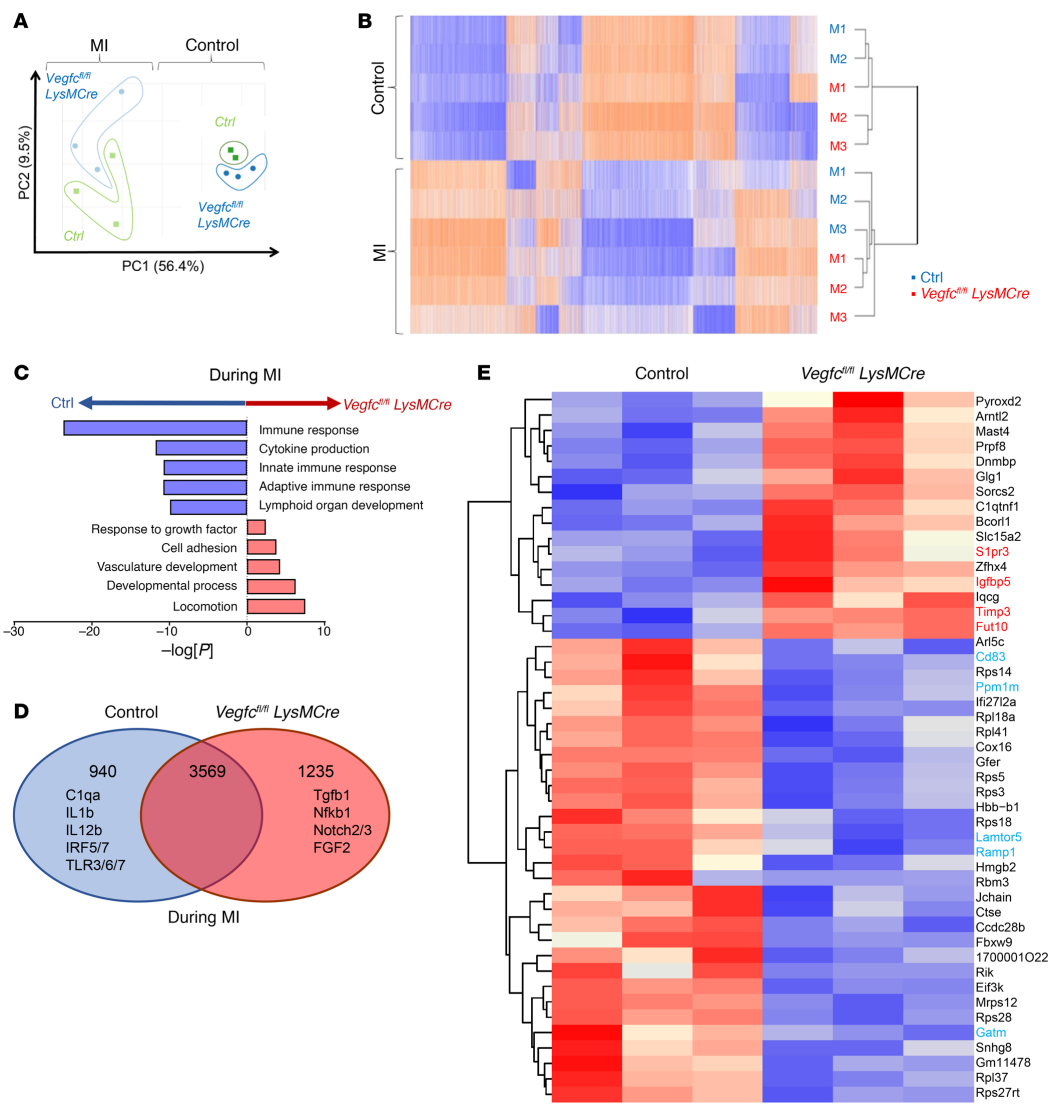
Impaired immune response with myeloid *Vegfc* deficiency after MI. Experimental C57BL/6 or *Vegfc^fl/fl^* versus *Vegfc^fl/fl^* *LysMCre* mice were subjected to coronary artery occlusion, and bulk mRNA gene expression analysis was performed for the LV. (**A**) Principal component analysis (PCA) revealing MI as a main source of variance in the data set. Data for the nonligated animals were clustered together, consistent with relatively comparable gene expression profiles at steady state. (**B**) Heatmap analysis and hierarchical clustering revealed distinct changes between nonligated and ligated animals at the transcriptional level. M1, M2, and M3 represent individual animals used for each group. (**C**) Gene ontology pathway interrogation revealed significant downregulation of immune response genes in the absence of myeloid *Vegfc*. In contrast, developmental pathways were induced in *Vegfc^fl/fl^* *LysMCre* mice, consistent with a hypertrophic response. (**D**) Venn diagram of differentially expressed or shared expression genes. (**E**) Heatmap of normalized top 50 absolute log fold changes in *Vegfc^fl/fl^* *LysMCre* mice compared with controls after MI. Genes highlighted in red are associated with inflammation and fibrosis, whereas those in cyan are associated with a lymphatic response and inflammation resolution.

**Figure 9:**
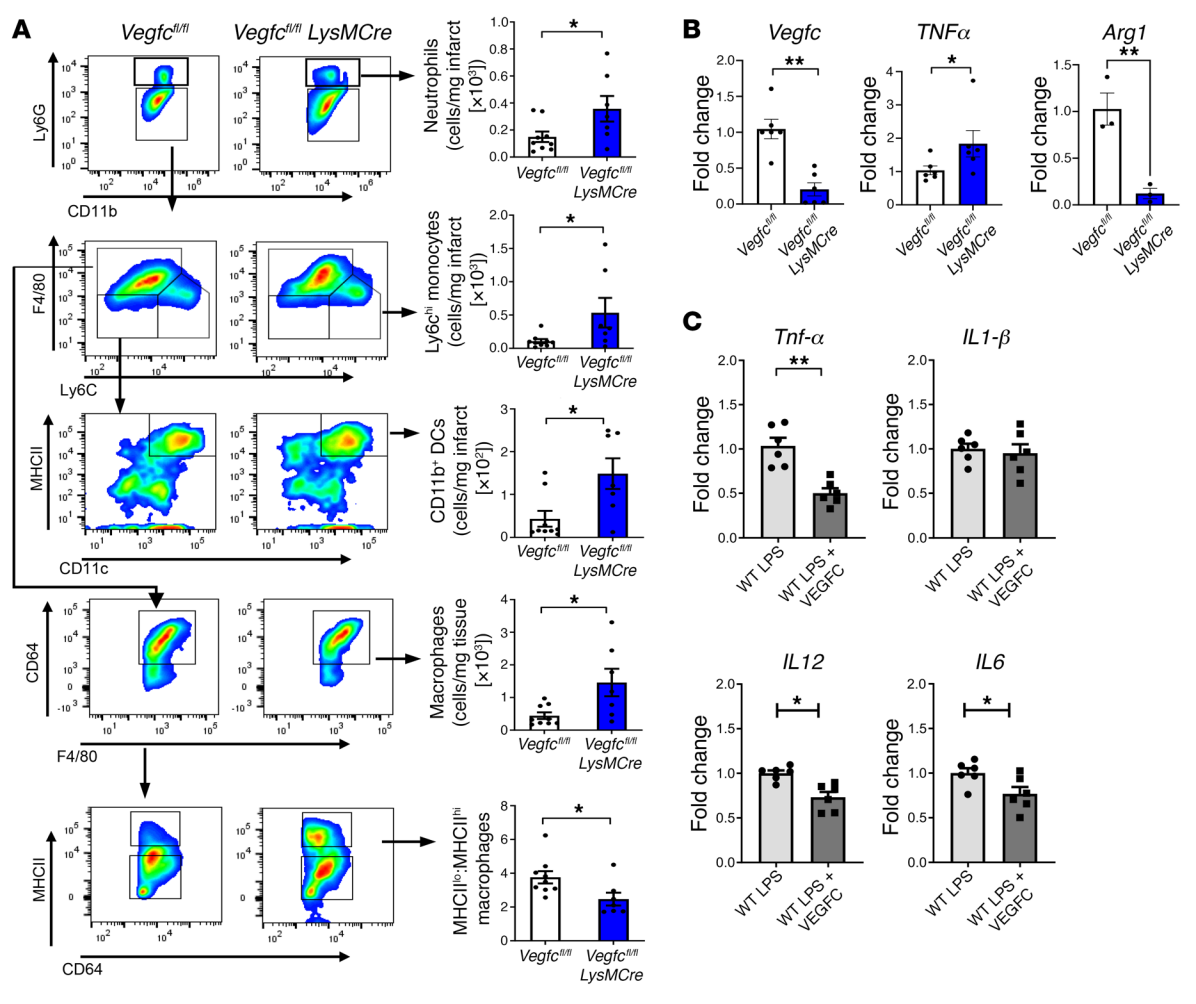
Evidence for heightened cardiac and macrophage inflammation in myeloid *Vegfc*–deficient mice. (**A**) Flow cytometric analysis of the ischemic AAR, 7 days after MI revealed heightened levels of CD11b^+^Ly6g^+^ neutrophils, Ly6c^hi^ monocytes, CD11b^+^CD11c^+^MHCII^hi^ DCs, and CD64^+^F4/80^+^ macrophages. Importantly, the ratio of MHCII^lo^ to MHCII^hi^ macrophages within the infarcted myocardium was significantly altered in *Vegfc*-deficient mice. *n =* 7–9 per group. *P < 0.05, by 2-tailed, unpaired *t* test. (**B**) BMDMs from *Vegfc^fl/fl^* and *Vegfc^fl/fl^* *LysmCre* mice were assessed for indicators of heightened inflammation. Transcript levels were measured by qPCR. An increase in markers of inflammation in *Vegfc*-deficient macrophages was observed along with reduced expression levels of *Arg1*. *n =* 3–6 per group. *P < 0.05 and **P < 0.004, by 2-tailed, unpaired *t* test. (**C**) VEGFC suppressed mRNA expression of inflammatory cytokines. qPCR of LPS-treated macrophages in culture treated with recombinant VEGFC versus control. *n =* 6 per group. *P < 0.05 and **P < 0.001, by 2-tailed, unpaired *t* test.

Next, we decided to further evaluate our findings in mouse models of conditional *Vegfc* loss of function. Interestingly, during the early reparative stage of experimental MI, mice deficient in *Vegfc* in myeloid cells (*Vegfc^fl/fl^* *LysMCre* mice) were more susceptible to scarring and had worsened cardiac function (Figure 7, A–C). Adverse remodeling was confirmed by histologic analysis of infarcted hearts from *Vegfc^fl/fl^* *LysMCre* mice, which had higher levels of fibrosis relative to *Vegfc^fl/fl^* control mice 28 days after MI (Figure 7C). Importantly, we confirmed that this difference in scarring was not due to alterations in acute infarct size, as seen by infarct and AAE measurements 24 hours after the I/R procedures (Supplemental Figure 6). To ascertain how *Vegfc* might affect inflammation resolution of the infarct, we performed mRNA-Seq of injured hearts 1 week after MI. Remarkably, gene ontology analysis identified a significant gene signature indicative of dysregulated inflammatory activation (Figure 8, A–D). More specifically, we found that the expression of genes associated with enhanced inflammation were increased in the *Vegfc^fl/fl^* *LysMCre* animals. Concurrently, these mice also showed reduced expression of genes associated with inflammation resolution and a lymphatic response to inflammation (Figure 8E). These data were reinforced by flow cytometry, which revealed increased inflammatory polarization of innate immune cells and cardiac macrophages (Figure 9A), even though earlier cardiac inflammation was unaffected (Supplemental Figure 7). Recent studies have shown that cardioprotective Tregs also accumulate in the infarcted heart (22, 23). Consistent with the hypothesis that myeloid *Vegfc* promotes inflammation resolution, we also detected lower levels of infarct-associated Tregs in *Vegfc^fl/fl^* *LysMCre* mice, while levels in the MLNs remained elevated (Supplemental Figure 8). Similarly, myeloid *Cd36–*deficient animals also maintained a propensity toward higher levels of inflammatory MHCII^hi^ macrophages within their infarcts compared with levels in control animals (Supplemental Figure 9). The gating strategies for these analyses are also provided in Supplemental Figure 10.

To strengthen our conclusions above, we performed pharmacological inhibition of the VEGFC receptor VEGFR3 using MAZ51 (24) each day for 3 days after MI. Our results showed a reduction of mCherry antigen uptake by cardiac and MLN macrophages (Supplemental Figure 11). To determine whether myeloid *Vegfc* might act to directly autoregulate macrophage function, for example through autocrine or paracrine mechanisms, we harvested primary bone marrow–derived macrophages (BMDMs) from *Vegfc^fl/fl^* *LysMCre* and *Vegfc^fl/fl^* mice and measured inflammatory cytokine production. As indicated in Figure 9B, *Vegfc*-deficient macrophages had elevated levels of proinflammatory *Tnfa* as well as reduced levels of arginase 1 (*Arg1*), with the latter found to be associated with alternative macrophage activation (25). Furthermore, direct administration of recombinant VEGFC reduced *Tnfa*, *Il6*, and *Il12* mRNA levels in LPS-activated macrophages (Figure 9C). Thus, macrophage-produced *Vegfc*, in addition to its contribution during cardiac lymphangiogenesis and antigen clearance to draining LNs, may also improve cardiac repair by directly suppressing excessive macrophage secretion of proinflammatory cytokines.

## Discussion

Taken together, our findings reveal a cardioprotective role for myeloid-produced VEGFC after cardiac injury. We also report that efferocytosis, as occurs after tissue injury, is a trigger for *Vegfc* induction. The underlying protective mechanisms are likely 2-fold. First, enhanced cardiac lymphangiogenesis has been reported to improve cardiac function after MI (9). Second, our findings argue that *Vegfc* deficiency in macrophages led to elevated production of proinflammatory cytokines. Macrophages in certain contexts may express the VEGFC receptor VEGFR3 (26) and therefore be susceptible to autocrine-mediated inflammatory regulation. In this scenario, antiinflammatory macrophage polarization has been linked to improved cardiac repair (27). Taken together, in Supplemental Figure 12, we propose a working model that integrates these findings.

Myeloid cells are known to contribute to vascular growth (28), including lymphatic signaling (18, 29), however, little is known regarding myeloid contributions to lymphatics in the adult heart. Also unclear are the potential triggers that activate the expression of lymphangiogenic factors by macrophages. Of relevance to cardiac ischemia, hypoxia-inducible transcription factors have been reported to coordinate lymphangiogenesis and VEGFC induction during wound healing (30). Here, we report macrophage efferocytosis as a VEGFC trigger. Beyond the removal of dead tissue, efferocytosis is also an important cellular reprogramming signal that induces cytokines and growth factors. These factors may act as autocrine or paracrine cues to activate tissue reparative cells, such as fibroblasts or endothelial cells, during tissue repair. For example, in the heart, efferocytosis is a trigger to produce myocardial VEGFA (7). When macrophages are triggered to synthesize growth factors for lymphatic endothelial cells, this in turn spatially focuses the lymphatic response at the site of tissue injury.

The scavenger receptor *Cd36* contributes to efferocytosis (31). CD36 has also been implicated in lymphangiogenesis, for example through monocytes, after binding the CD36 ligand thrombospondin 1 (32), as well as in the regulation of lymphatic junction integrity through the modulation of *VEGFR2/AKT* signaling in lymphatic endothelial cells (33). Since CD36 has no inherent enzymatic function, future studies are necessary to determine which kinases CD36 directly engages to activate the transcription of *Vegfc*, or, alternatively, whether the process of AC engulfment per se leads to a common *Vegfc* transcriptional trigger. Another potential mechanism by which CD36 might signal in macrophages is through its function as a transporter of fatty acid metabolites. For example, *Cd36* has been implicated in endothelial metabolism (34, 35) and angiogenesis (36).

Despite its most notable vascular roles, *Vegfc* has also been linked to noncanonical roles of immunomodulation. For example, evidence exists that innate immune cells express VEGF receptors including VEGFR3 (26) and that VEGF signaling controls hematopoiesis (37). Moreover, a VEGFC and VEGFR3 axis regulates macrophage plasticity in association with the amelioration of experimental inflammatory bowel disease (38). Furthermore, a recent report suggested that VEGFC/VEGFR3 signaling promotes macrophage efferocytosis in the acute lung injury model (39). Consistent with these findings, we observed reduced cardiac antigens in the macrophages of animals administered a VEGFR3 inhibitor. Future studies are necessary to understand how VEGFC and growth factors derived from lymphatic endothelial cells may affect macrophage reprogramming and function.

Like most studies, the conclusions of our experiments have limitations. For example, *LysMCre* may delete *Vegfc* in neutrophils. However, our data indicate that *Vegfc* was not expressed in cardiac Ly6g^+^ neutrophils (Figure 3). We also cannot discount the contribution of *Vegfc* from other sources such as blood endothelial cells. Our data are also consistent with the notion that efferocytosis, because of its critical role in maintaining tissue homeostasis, engages redundant or overlapping pathways to prevent secondary necrosis of ACs. In addition, risk factors for MI include metabolic disease and obesity, the latter of which is also linked to defective lymphatics (40), as well as increased inflammation (41). Thus, it will be of clinical relevance to explore the role of cardiac lymphatics and inflammatory cell crosstalk during experimental MI, in the setting of hyperlipidemia and obesity. For example, hyperlipidemic atherosclerosis is associated with decreased lymphangiogenesis and an impaired ability of CD11b^+^ DCs to dampen inflammation due to decreased emigration to regional LNs (42). Although we did not directly characterize the phenotype of mCherry^+^ phagocytes in the MLNs, we can argue that most likely they will dampen inflammation. This is especially important, considering the systemic inflammatory effects a MI might have.

Taken together, our studies identify cardiac macrophages as a significant stimulus of *Vegfc* in the inflamed heart. Efferocytosis, such as occurs during tissue injury, is a trigger for macrophage expression of *Vegfc*, and myeloid *Vegfc* is necessary to ameliorate the progression to heart failure after MI. Studies are underway to elucidate basic molecular mechanisms by which efferocytosis triggers *Vegfc* induction and *Vegfc*-dependent macrophage polarization, as well as the potential clinical targeting of these pathways after cardiac ischemia.

## Methods

### Animals.

*Cd36^–/–^* mice have been described previously (43) and were obtained from Columbia University (New York, New York, USA). *Cd36^fl/fl^* mice (44) were generated in-house. *Vegfc^fl/fl^* mice have been previously described (45) and were generated in-house. Conditional *Vegfc* GOF mice have been described previously (46) and are referred to herein as *VegfcGOF* mice. Briefly, the full-length cDNA of mouse *Vegfc*, including a V5 tag, was inserted into the first intron of the *Eif1a* locus. Vegfc expression was prevented by a floxed neomycin triple poly(A) cassette preceding *Vegfc*. These mice were crossed with *LysMCre* mice obtained from The Jackson Laboratory to induce myeloid expression of *Vegfc*. α*MHC-mCherry* B6 D2-tg mice were obtained from The Jackson Laboratory. *LysMCre EGFP* mice were obtained from Ronen Sumagin (Feinberg School of Medicine, Northwestern University, Chicago, Illinois, USA), as previously described (47). *C57BL/6* and *Prox1Tdtomato* mice were obtained from The Jackson Laboratory.

### Materials, antibodies, and oligonucleotides.

Macrophage VEGFC immunoblotting was performed as previously described (29). Plasma was collected from mice, and commercially available ELISA kits were used to measure the circulating levels of VEGFC (Cusabio Technology) as previously described (48). qPCR assays for *Vegfc* were carried out using SYBR Green Mastermix qPCR reactions (Bio-Rad Laboratories) as described previously (48). Relative *Vegfc* mRNA levels were calculated using the 2ΔΔCt method. Additional reagents are listed in Supplemental Table 1. Primer sequences are listed in Supplemental Table 2.

### Coronary artery ligation.

Permanent ligation MI surgeries were performed as previously described (6). In brief, mice were anesthetized with Avertin and received buprenorphine (0.1 mg/kg, s.c.) prior to surgery. Mice were secured in a supine position, intubated endotracheally, and ventilated with an Inspira Advanced Safety Single Animal Pressure/Volume Controlled Ventilator (Harvard Apparatus). The chest wall was shaved and sterilized with povidone iodide and alcohol prep pads. With the aid of a dissecting microscope, a left thoracotomy was performed to expose the left ventricle (LV). The LAD coronary artery was visualized and permanently ligated with a 7-0 monofilament nylon suture at a site 2 mm distal to its emergence from under the left atrium. MI was verified by the onset of blanching or pale discoloration of the anterior wall of the LV. The surgical site was closed in layers, starting with the chest wall, finishing with skin and subcutaneous tissue, using 6-0 monofilament nylon sutures. Following surgery, the mice received buprenorphine (0.1 mg/kg, s.c.) every 12 hours and up to 48 hours for pain management. The I/R procedures were carried out as described above, with the exception that the LAD artery was temporarily ligated for 45 minutes followed by reopening of the ligature to allow for reperfusion. Mice that succumbed to surgery within 48 hours of MI surgery were treated as technical errors and excluded from the analyses.

### Infarct and AAR measurements.

The mice were anesthetized, intubated endotracheally, and ventilated with an Inspira Advanced Safety Single Animal Pressure/Volume Controlled Ventilator (Harvard Apparatus). With the aid of a dissecting microscope, the thoracic cavity was opened to expose the heart. Using a 30 gauge insulin needle, 50 μL FluoSpheres Polystyrene Microspheres (Invitrogen, Thermo Fisher Scientific, red fluorescent 580/605) was injected into the LV. Hearts were excised 1 minute later and sectioned into 1 mm coronal slices using a Mouse Heart Slicer Matrix (Zivic Instruments). Infarcted tissue and viable myocardium were visualized by staining the slices with 1% 2,3,5-triphenyltetrazolium chloride (TTC) (MilliporeSigma) in saline. The AAR was visualized by placing the slices under a fluorescence microscope. Both the infarct and the AAR were measured as a percentage of the LV using ImageJ (NIH). Infarct size, expressed as a percentage of the AAR, was calculated by dividing the sum of the infarct areas from all sections by the sum of the AAR from all sections and multiplying by 100.

### Echocardiography.

Cardiac function was assessed by transthoracic echocardiography on anesthetized mice before and on day 28 after MI using a Vevo 3100 equipped with a 25 MHz probe (VisualSonics) as described previously (6). Parasternal short-axis images were acquired using M-mode 1 mm before, at, and after the papillary muscles. Image analysis was performed using Vevo LAB 3.1 software (VisualSonics). Measurements of the left ventricular internal dimension at both end-systole and end-diastole were made in 3–6 consecutive cardiac cycles and averaged for analysis. In a subset of animals, 1 long-axis view and 3 short-axis B-mode views (measured near the apex, papillary muscle, and distal areas of the heart) were used to trace end-diastolic and end-systolic areas to determine left ventricular volume. Left ventricular volumes and the left ventricular EF were then calculated using a modified Simpson’s method.

### Flow cytometry.

Mice were euthanized and hearts were extensively flushed with saline to remove peripheral cells. Infarcted myocardium was then excised, minced with fine scissors, and digested with collagenase and DNase at 37°C for 30 minutes as described previously (21). Cardiac tissue was then homogenized by pipetting and subsequently filtered through a 40 μm cell strainer. Erythrocytes were lysed, and total viable cell numbers were determined by Trypan blue staining. Cells were then incubated with Fc Block (BioLegend) for 15 minutes before being labeled with fluorescently conjugated antibodies for 30 minutes. Flow cytometry was performed on a LSRFortessa X-20 cytometer (BD Biosciences), and data were analyzed by FlowJo software (TreeStar). Macrophages were identified as CD11b^+^Ly6GLy6C^lo^F4/80^+^ and further distinguished by MHCII expression (Supplemental Figure 10). Monocytes were identified as CD11b^+^Ly6G^lo^Ly6C^hi^F4/80^–^, and DCs were further distinguished by CD11c and MHCII expression. Neutrophils were identified as CD11b^+^Ly6G^+^.

### qPCR.

RNA was extracted from cell culture, infarcted myocardium, or sorted macrophages using TRIzol (Invitrogen, Thermo Fisher Scientific) according to the manufacturer’s instructions. mRNA was transcribed to cDNA using the iScript cDNA synthesis kit (Bio-Rad). qPCR was performed on an Applied Biosystems StepOnePLus System (Thermo Fisher Scientific) using SYBR Green probes. Results are expressed as ΔΔCt values normalized to β-2m and graphed as relative expression compared with controls.

### Histology and immunofluorescence staining.

Murine hearts were flushed extensively with PBS and then infused and fixed with 4% phosphate-buffered formalin at physiological pressures. For immunohistochemistry, frozen section samples were acclimated in OCT and then placed in cryomolds and submerged in fresh OCT. Blocks were flash-frozen in an isopentane slurry and chilled with liquid nitrogen. Transverse cryosections were cut at a thickness of 10 μm on a Leica cryostat and placed on Superfrost Plus–coated slides (Thermo Fisher Scientific). Before antibody staining, sections were rehydrated with PBS and permeabilized with ice-cold methanol, followed by nonspecific protein binding blocked with 1% albumin for 1 hour. Incubation with the indicated primary antibodies was carried out overnight at 4°C. After rinsing with saline, the sections were incubated with the respective fluorescence-conjugated secondary antibodies and counterstained with DAPI nuclear stain before sealing with VECTASHIELD Antifade Mounting Medium (Vector Laboratories). Slides were visualized under a fluorescence scope, and images were processed using ImageJ.

### Bone marrow macrophage isolation.

Briefly, mice were euthanized by CO_2_ asphyxiation, and the hind limbs were removed with surgical scissors. Femurs and tibiae were extracted and placed on ice in sterile PBS. In a biological cabinet, the bones were briefly sterilized in 70% ethanol followed by flushing using a 10 mL syringe/26 gauge needle filled with DMEM, and the contents were collected in 15 mL conical tubes. Following erythrocyte lysis, the cell suspension was filtered and plated at a density of 2 × 10^6^ cells per 100 mm Petri dish in macrophage media (DMEM, supplemented with a 100 U/mL penicillin/streptomycin mixture, 10% FBS, 1% sodium pyruvate, 1% glutamine, and 20% L-929 conditioned media). After 4 days, 50% of the medium was replaced and then completely replaced after 6 days. The medium was again replaced prior to experimental use.

### Efferocytosis assays.

BMDMs were incubated with 100 ng LPS (MilliporeSigma) in complete media (DMEM, 100 U/mL penicillin/streptomycin mixture, 10% FBS, 1% sodium pyruvate, 1% glutamine) for the indicated durations, and cell lysates were collected for further analysis as described below. For assessment of efferocytosis, thymuses and spleens were harvested from 6-week-old mice using sterile techniques and cultured in complete media. Apoptosis was induced by the addition of 100 ng/mL dexamethasone in thymocytes for 4–6 hours. For splenocytes, apoptosis was induced by UV irradiation for 8 minutes followed by 2 hours of culturing at 37°C. Apoptosis was confirmed using a flow cytometric annexin V staining protocol. Washed and counted ACs were then cocultured with BMDMs at a ratio of 5:1 for the indicated duration and medium, and cell lysates were collected for Western blotting and RNA assessment.

### RNA-Seq and ChIP-Seq.

For bulk tissue RNA-Seq analysis, infarcted left ventricular tissue was collected from *Vegfc^fl/fl^* *LysMCre* mice and *LysMCre* mice 9 days after MI. In naive animals, LVs of the same approximate mass and anatomical area were also collected. RNA was extracted by column-based, solid-phase extraction using the RNeasy Plus Mini Kit (QIAGEN), and quality and quantity were assessed using the Agilent Bioanalyzer (Agilent Technologies) and a Qubit Fluorometer (Invitrogen, Thermo Fisher Scientific), respectively. Libraries were generated using a TruSeq Stranded mRNA kit (Illumina) according to the manufacturer’s protocol, and the library quality was assessed with a Bioanalyzer. Sequencing was performed using the Illumina HiSEQ 4000 according to the manufacturer’s provided protocols and reagents. Sequencing and differential gene expression analysis were performed at the Northwestern Center for Genetic Medicine NUseq Core. For CHIP-Seq, primary macrophages were collected from C57BL/6J mice and cocultured with apoptotic Jurkat cells (compared with controls with no ACs). DNA was cross-linked, sheared, and then co-immunoprecipitated with an anti-H3K27Ac antibody. CHIP peak calling and downstream analysis were carried out using HOMER software (49). A heatmap was generated using EaSeq (50). The RNA-Seq and Chip-Seq data sets included in this study are available in the NCBI’s Gene Expression Omnibus (GEO) database (GEO GSE196867).

### Protein analysis.

Cells were lysed in RIPA buffer with a protease inhibitor cocktail. Protein extracts were subjected to SDS-PAGE. Anti-VEGFC (Santa Cruz Biotechnology) and anti–β-actin antibodies were used for immunodetection overnight at 4°C. Following exposure to HRP-conjugated secondary antibodies, reactive bands were visualized with enhanced ECL plus reagents, and band density analyzed with ImageJ. See complete unedited blots in the supplemental material. 

### Statistics.

Analyses were performed with GraphPad Prism 8 (GraphPad Software). Comparisons between 2 groups were done using a 2-tailed, unpaired *t* test with a 95% CI. For comparisons of more than 2 variables, 1-way or 2-way ANOVA was used with a 95% CI, and when necessary, Tukey’s test was used to correct for multiple comparisons. The experimental sample size is indicated in the figures and represents pooled data from 2 or more independent experiments. Data are presented as the mean ± SEM. The criteria for significant differences are indicated in the figure legends. Statistical significance for all figures was set at *P < 0.05, **P < 0.01, ***P < 0.001, and ****P < 0.0001.

### Study approval.

All animal studies were conducted in accordance with guidelines using protocols approved by the IACUC of Northwestern University.

## Author contributions

KEG, WM, GO, and EBT designed the experiments and co-wrote the manuscript. KEG and WM performed most of the experiments, and LG, XL, and MD collaborated on some of them. KEG carried out surgical procedures, and CL assisted with bioinformatics analysis. MK provided the *Vegfc^fl/fl^* mouse strain, and MF provided the *Cd36^fl/fl^* mouse strain.

## Supplementary Material

Supplemental data

## Version 1 03/10/2022

In-Press Preview

## Version 2 05/02/2022

Electronic publication
