# Supplementary material for: Macrophage-produced VEGFC is induced by efferocytosis to ameliorate cardiac injury and inflammation
Source: J Clin Invest. 2022 May 2;132(9):e140685. doi: 10.1172/JCI140685 (PMC9057589; doi:10.1172/JCI140685)
Supplement: Supplemental data [file jci-132-140685-s089.pdf]

| For Flow cytometry | | | |
|---|---|---|---|
| **Antibody Target** | **Dilution** | **Clone** | **Supplier** |
| mCD3 | 1:200 | 17A2 | BD Bioscience |
| mCD4 | 1:200 | GK1.5 | Invitrogen |
| mCD8 | 1:200 | 53-6.7 | Biolegend |
| mCD11b | 1:200 | M1/70 | Biolegend |
| mCD11c | 1:200 | N418 | Biolegend |
| mCD19 | 1:200 | 115508 | Biolegend |
| mCD25 | 1:200 | PC61.5 | eBioscience |
| mCD45 | 1:200 | 30-F11 | Biolegend |
| mCD64 | 1:200 | X54-5/7.1 | Biolegend |
| mF4/80 | 1:200 | BM8 | Biolegend |
| mFOXP3 | 1:200 | MF-14 | Biolegend |
| mLy6C | 1:200 | HK1.4 | Biolegend |
| mLy6G | 1:200 | 1A8 | BD Bioscience |
| MHCII I-A/I-E | 1:200 | M5/114.15.2 | Biolegend |
| mCCR2 | 1:200 | FAB5538A | R&D Systems |
| mTim4 | 1:200 | F31-5G3 | Biolegend |
| mVegf-C | 1:200 | E-6 | SCBT |
| Zombie Aqua Fixable Viability Kit | 1:1000 | Cat#: 423101 | Biolegend |
| **For Immunofluorescence/Western Blotting** | | | |
| **Antibody Target** | **Dilution** | **Clone/Catalog #** | **Supplier** |
| Goat anti-mouse IgG A594 | 1:500 | Poly4053 | Biolegend |
| Goat anti-mouse IgG A488 | 1:500 | Poly4053 | Biolegend |
| Donkey anti-goat TR | 1:500 | SAB3700320 | MilliporeSigma |
| Anti-Lyve1 | 1:200 | BAF2125 | R&D Systems |
| CD68 | 1:200 | FA-11 | Biolegend |
| PECAM | 1:1000 | 553370 | Pharmingen |
| pStat6 | 1:1000 | Cat#: mab56554 | Cell Signaling |
| Stat6 | 1:1000 | mab5397 | Cell Signaling |
| Beta Actin-HRP | 1:1000 | 2F1-1 | Biolegend |
| **Chemicals/Recombinant Proteins/Commercial Kits** | | | |
| RNAeasy Plus mini kit | Cat#: 74034 | | Qiagen |
| TruSeq® Stranded mRNA Library Prep | Cat#: 20020594 | | Illumina |
| Anti-Histone H3 (acetyl K27) | ab4729 | | Abcam |
| Mouse Vegfc ELISA Kit | Cat#: CSB-E07361m | | Cusabio |
| Cytochlasin D | Cat#: C8273-1MG | | MilliporeSigma |
| Etomoxir | Cat#: E1905 | | MilliporeSigma |
| Lipopolysaccharide from E. coli O111:B4 | Cat#: L4391 | | MilliporeSigma |
| RBC Lysis Buffer (10X) | Cat#: 420301 | | BioLegend |
| Neomycin solution | Cat#: N1142 | | MilliporeSigma |
| 2,3,5-Triphenyltetrazolium chloride | Cat#: T8877 | | MilliporeSigma |
| MAZ51 | Cat#: 06-127 | | MilliporeSigma |
| Sirius Red (Direct Red 80) | Cat#: 365548-5G | | MilliporeSigma |
| FluoroSpheres (580/605) | Cat#: F8834 | | ThermoScientific |
| Collagenase Type 2 | Cat#: LS004177 | | Worthington |

**Supplemental Table I.**

| Primer Lists | |
|---|---|
| **Target** | **Sequence** |
| *Vegf-c* | Vegfc-F: TCC CCT GTC CTG GTA TTG AG<br>Vegfc-R: CGA GGT CAA GGC TTT TGA AG |
| *Tnf alpha* | Tnf-F: ACG GCA TGG ATC TCA AAG AC<br>Tnf-R: AGA TAG CAA ATC GGC TGA CG |
| *IL1Beta* | Il1b-F: TAC GGA CCC CAA AAG ATG A<br>Il1b-R: TGC TGC TGC GAG ATT TGA AG |
| *IL6* | Il6-F: GCC TTC TTG GGA CTG ATG CT<br>Il6-R: TGC CAT TGC ACA ACT CTT TTC |
| *IL12* | mIL12-F: ACGAGAGTTGCCTGGCTACTAG<br>mIL12-R: CCTCATAGATGCTACCAAGGCAC |
| *Arg1* | Arg1-F: ATGGAAGAGACCTTCAGCTAC<br>Arg1-R: GCTTTCCCAACAGTTGGG |
| *B2M* | B2m-F: CTG CTA CGT AAC ACA GTT CCA CCC<br>B2m-R: CAT GAT GCT TGA TCA CAT GTC TCG |

**Supplemental Table II.**

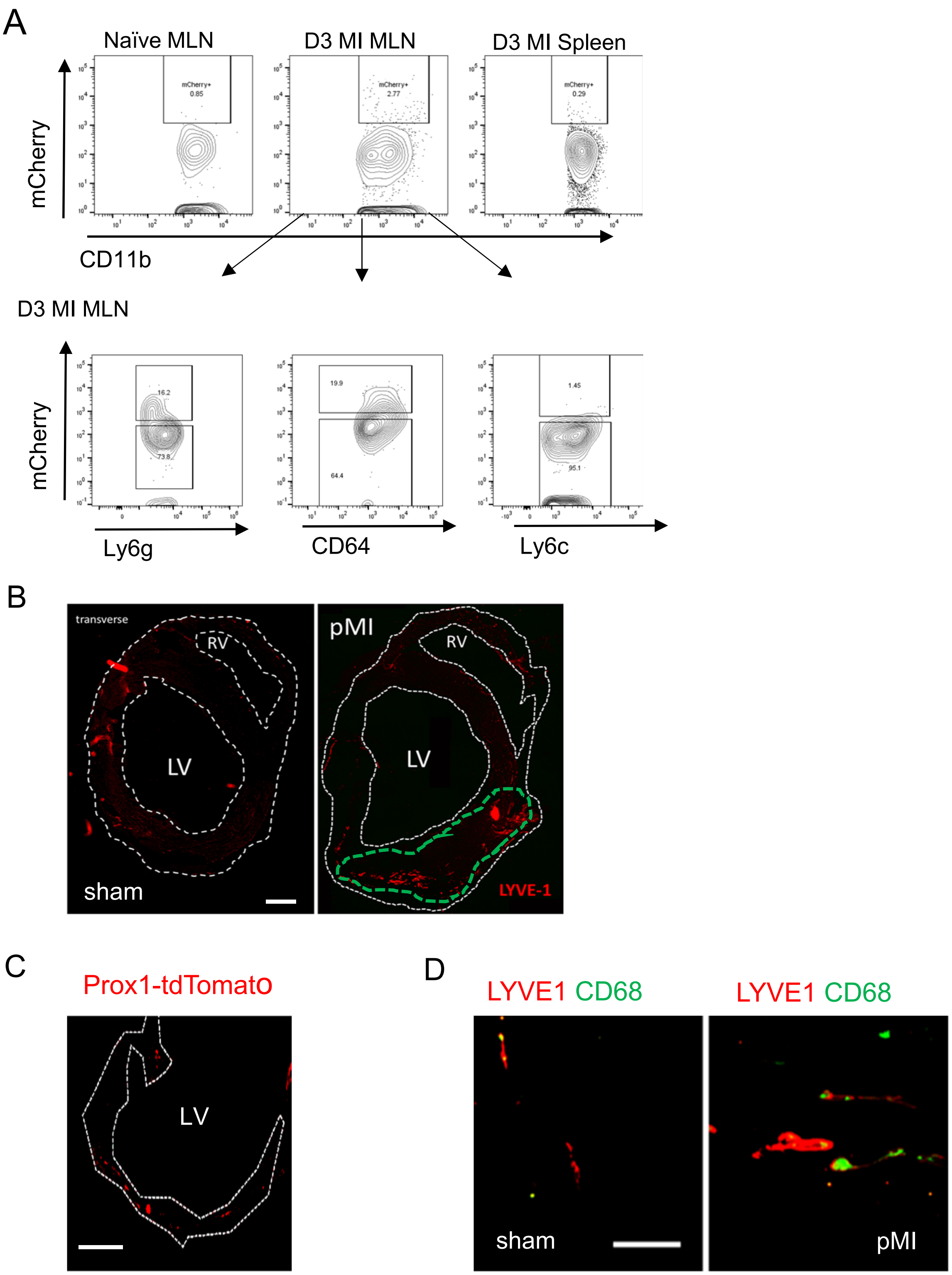


**Supplemental Figure 1. Experimental myocardial infarction (MI) induces markers of cardiac lymphangiogenesis.** (**A**) Representative flow cytometric analysis of MLNs from mCherry mice prior to and post LAD ligation. Top panel shows mCherry antigen uptake by CD11b+ cells in mediastinal lymph nodes and spleen 3 days post MI. Bottom panel shows mCherry antigen uptake 3 days post MI by $Ly6g^{+}$ neutrophils, $CD64^{+}$ $F4/80^{+}$ macrophages and $Ly6c^{hi}$ monocytes. (**B**) Left anterior descending (LAD) arteries of C57BL/6J adult mice were ligated and transverse cross sections stained by fluorescent immunohistochemistry for LYVE1 (lymphatic vessel endothelial receptor 1) tubular staining post MI (pMI) versus sham surgery control. White dotted lines circumscribe the heart and LV indicates left ventricle while RV indicates right ventricle. Green-dotted line indicates ischemic area at risk. Images taken of hearts at ~7 days post MI. (Scale bar = 1mm) (**C**) Prox1-tdTomato mice were subjected to ligation of the LAD and *Prox1Tdtomato* images acquired at the circumscribed infarct area at risk by fluorescent microscopy. (Scale bar = 1mm) (**D**) Tubular LYVE1+ CD68-negative tubular staining in C57BL/6J sham mice versus LAD-ligated mice. (Scale bar = 100µm).

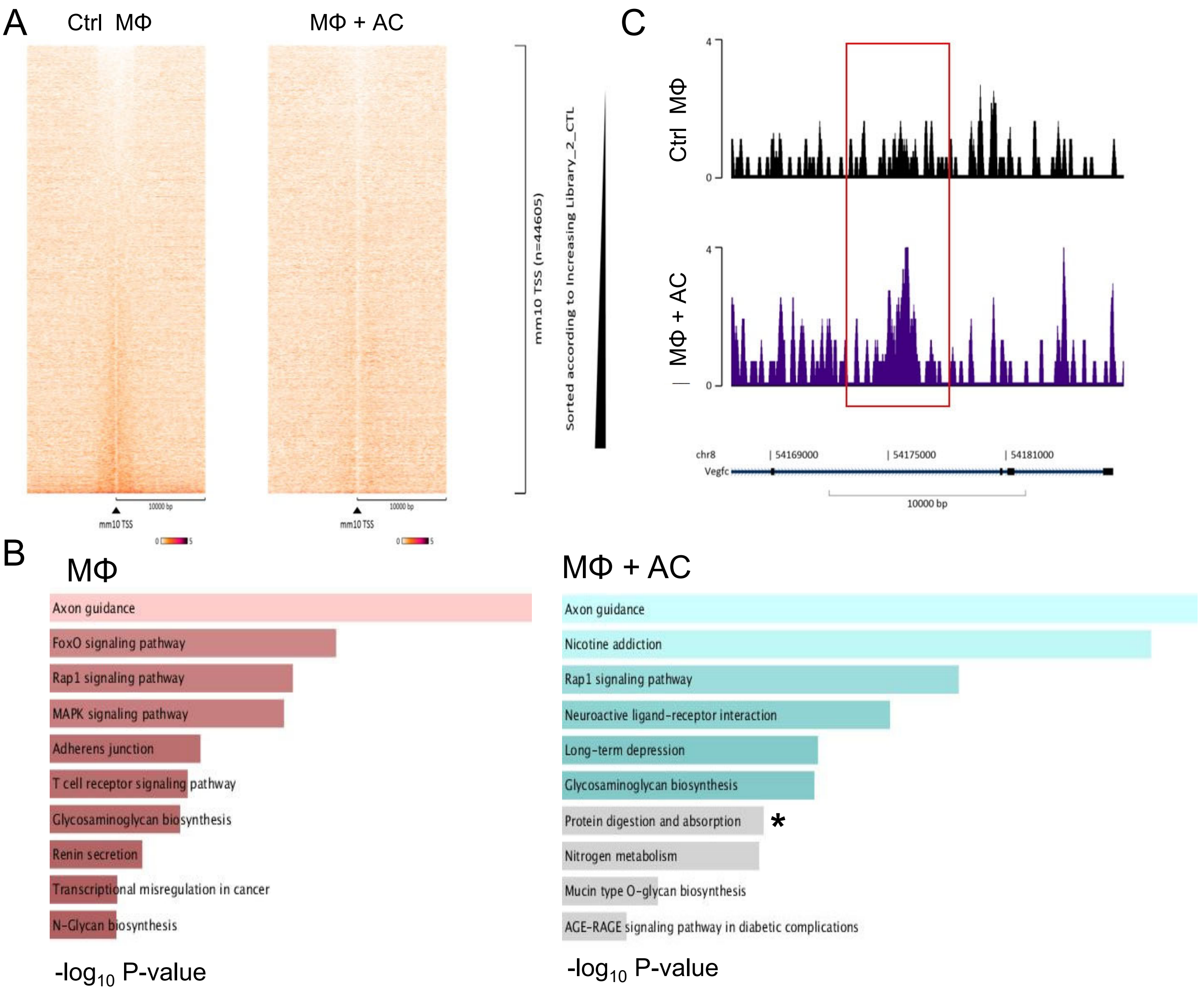


D

| footprintDB template | Source | Organisms | STAMP e-value | Motif similarity | footprinDB PWM / Consensus |
|---|---|---|---|---|---|
| PB0166.1: Sox12_2 | JASPAR 2016 | Mus musculus | 2.5e-04 | 6.60 / 10 | -----tTkCTcwMwG- awtyCTTTGTstrwkk |
| M0310_1.02: Cebpe / T025966_1.02 | CISBP 1.02 | Mus musculus | 6.5e-04 | 5.30 / 8 | tTkCTcwMwG TTwyGCAA-- |
| MA0520.1: Stat6 | JASPAR 2016 | Mus musculus | 7.4e-04 | 6.33 / 10 | -tTkCTcwMwG---- rkTTCtswrGAArws |

**Supplemental Figure 2. Epigenetic screen for open chromatin accessibility mark Histone3K27Acetylation (H3K27Ac) after efferocytosis implicates transcriptional activation of *Vegfc* and open transcription factor binding site footprints.** Primary macrophages were cultured without (Ctrl) versus with apoptotic cells (AC) and subjected to chromatin immunoprecipitation sequencing (ChIP-seq) for H3K27ac. **(A)** Mus musculus (mm10) genome assembly heatmap of H3K27ac ChIP-seq signal at transcription start sites (TSS) across the mouse genome. Analyzed with University of California Santa Cruz Genome Browser. (**B**) KEGG pathway analysis of genes associated with the top 3000 peak score regions. Notable enrichment for “protein digestion and absorption” in apoptotic cells-treat group. (**C**) Evidence of increased chromatin accessibility at sites within the *Vegfc* locus. (**D**) Transcription factor footprint analysis at open chromatin sites during efferocytosis.

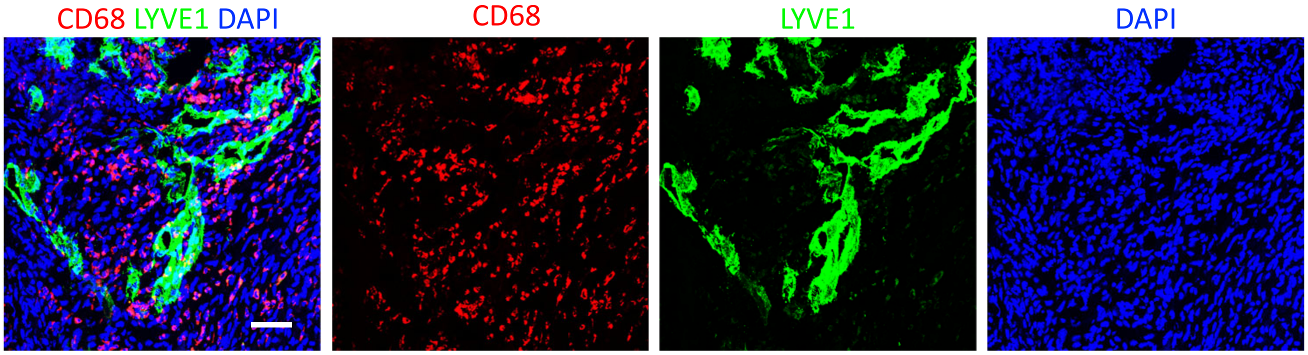


**Supplemental Figure 3. Juxtaposition of cardiac CD68 positive macrophage cells with LYVE1 lymphatic cells.** LYVE1+ lymphatic endothelial cell (LEC) cells are stained green, and $CD68^{+}$ macrophage cells are stained red. Images were taken from cardiac sections after coronary artery ligation of experimental mice at ~1week post MI. Scale bar = 50μm.

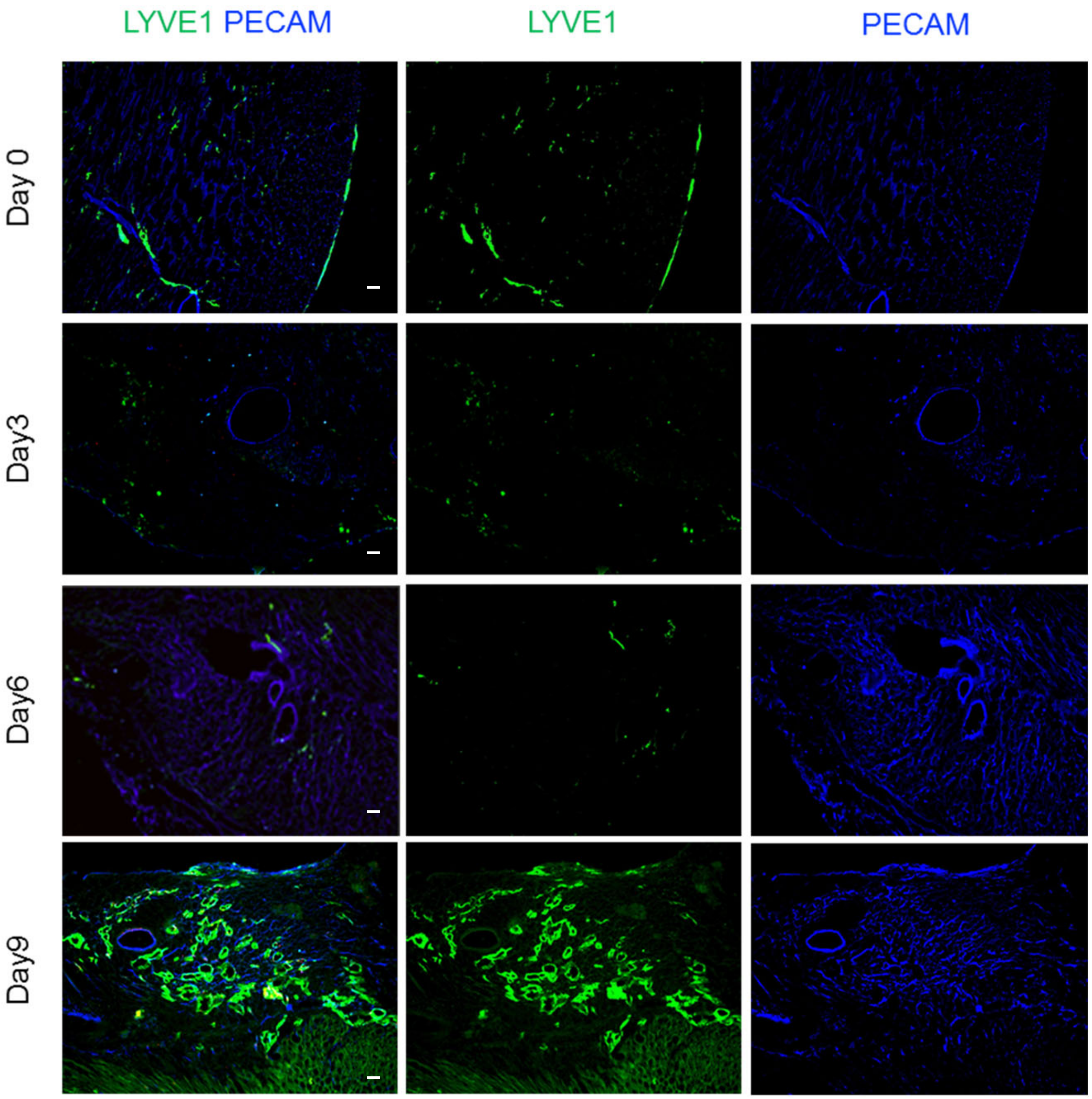


**Supplemental Figure 4. Time course of LYVE1 staining after ischemia then reperfusion (I/R) in mice.** Myocardial Immuno-fluorescent staining of indicated markers in transverse cross-sections of mice after cardiac I/R. Times are days post I/R. Scale bar = 50 µm.

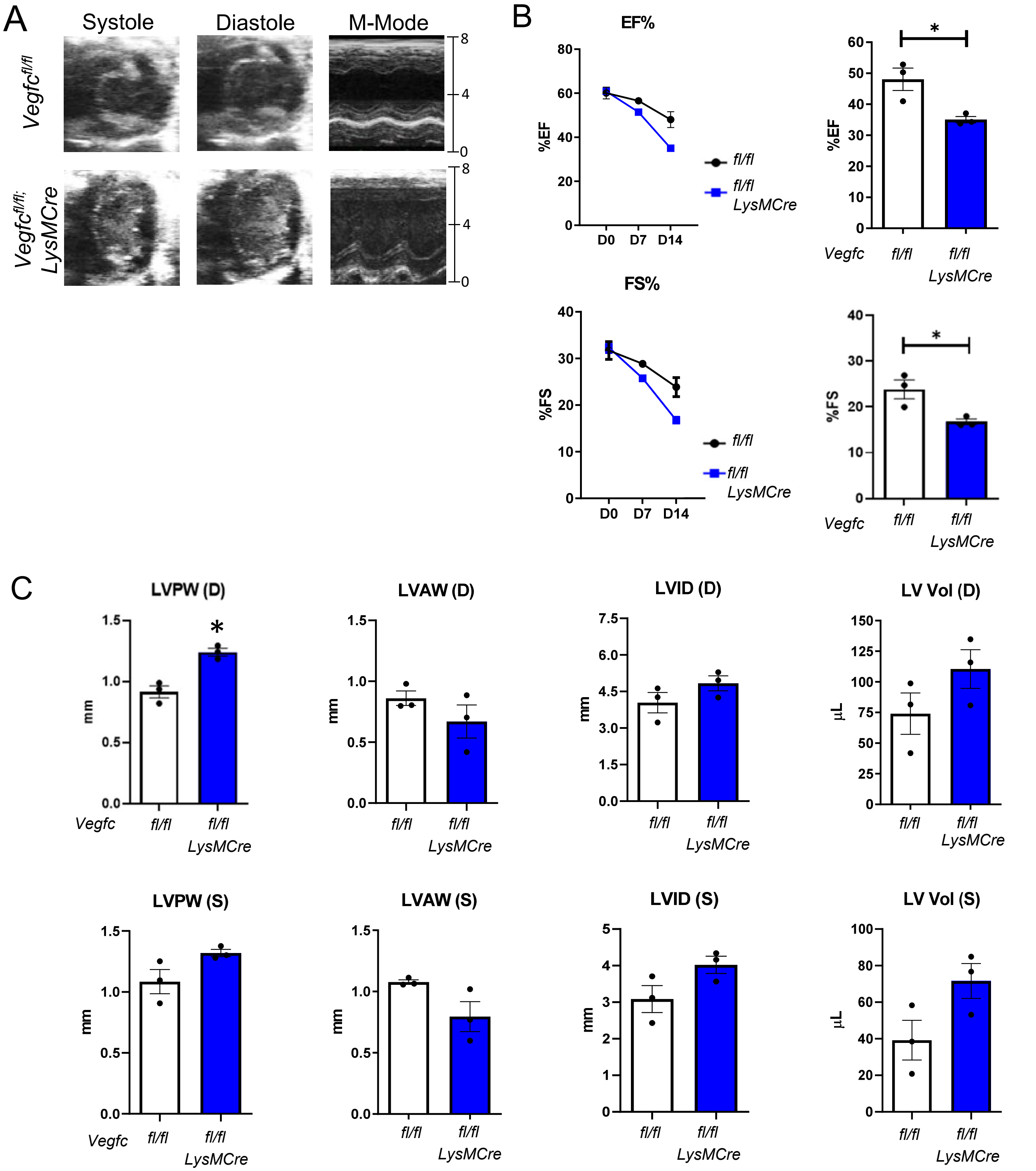


**Supplemental Figure 5. Myeloid *Vegfc* deficiency leads to impaired cardiac function after reperfusion of ischemic hearts.** (**A**) *Vegfc*$^{fl/fl}$*;LysMCre* mice along with littermate controls were subjected to LAD ligation followed by reperfusion (ischemia reperfusion I/R). Parasternal short-axis M-mode measurements were collected at prior to surgery (D0) and again at D14 post IRI procedure. (**B**) Time course of cardiac function between experiment groups at indicated days (D) post I/R per %EF and %FS as measurements of cardiac function. n = 3/group. * $p < 0.05$. (**C**) Parameters gained from parasternal M-mode measurements describing degree of anterior wall thinning, ventricular diameter, and volume in *Vegfc*-deficient animals after I/R. (d) Diastole, (s) Systole. n = 3/group. * $p < 0.05$.

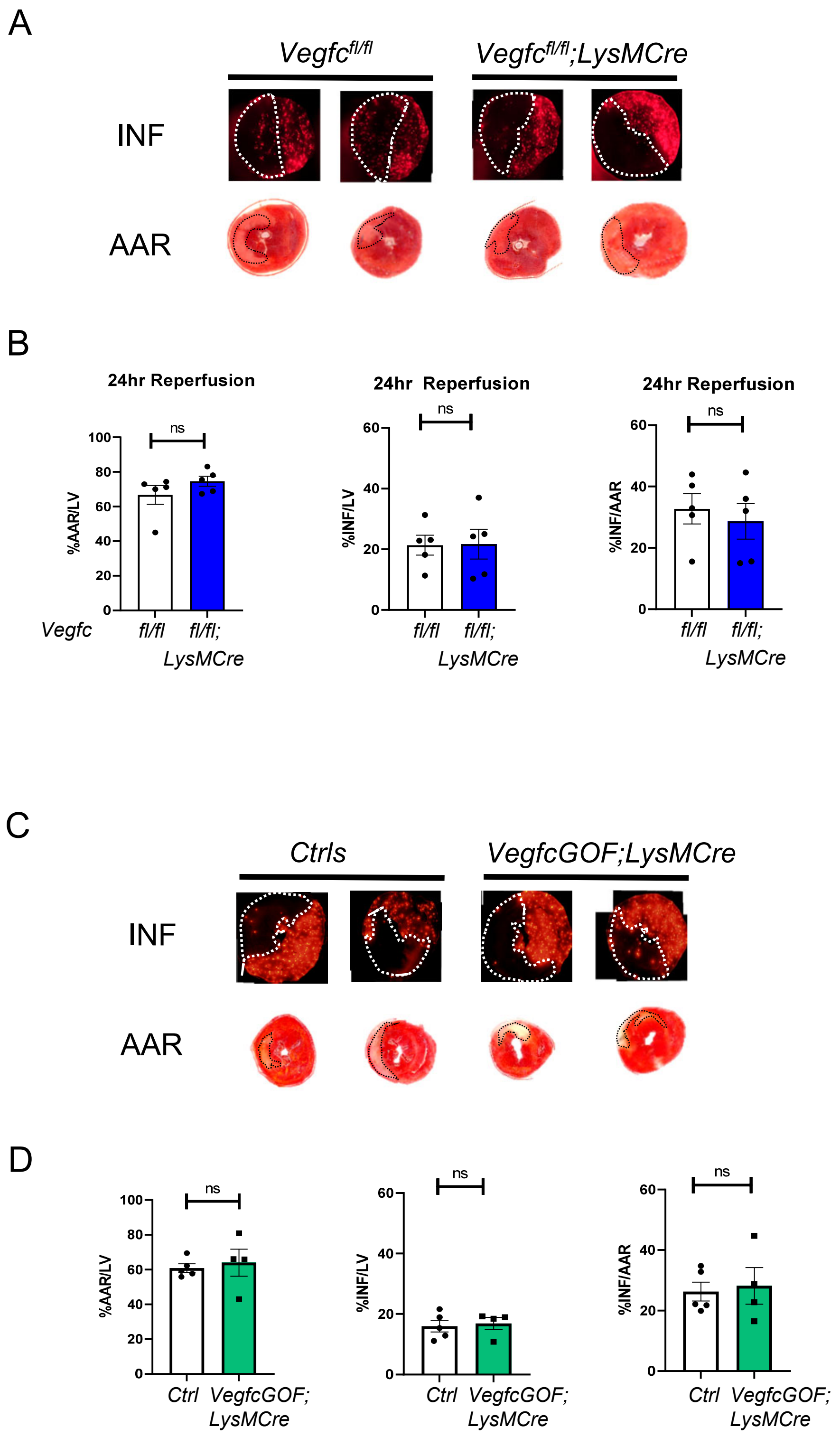


**Supplemental Figure 6. Myeloid-derived VEGFC does not affect infarct size 24h post I/R. (A)** *Vegfc^fl/fl^* and *Vegfc^fl/fl^;LysMCre* mice were subjected ischemia then reperfusion (I/R). Area-at-risk (AAR) was determined by intramyocardial circulation of fluorescent microbeads, while infarct (INF) size was determined by TTC staining after 24hrs reperfusion and quantified in (**B**) n = 5 mice per group. NS – No significance **(C)** In a similar fashion, infarct and area-at-risk measurements were obtained in *VegfcGOF;LysMcre* and littermate controls and quantified in (D). N = 4-5 mice per group. No significant difference in acute injury were observed between groups.

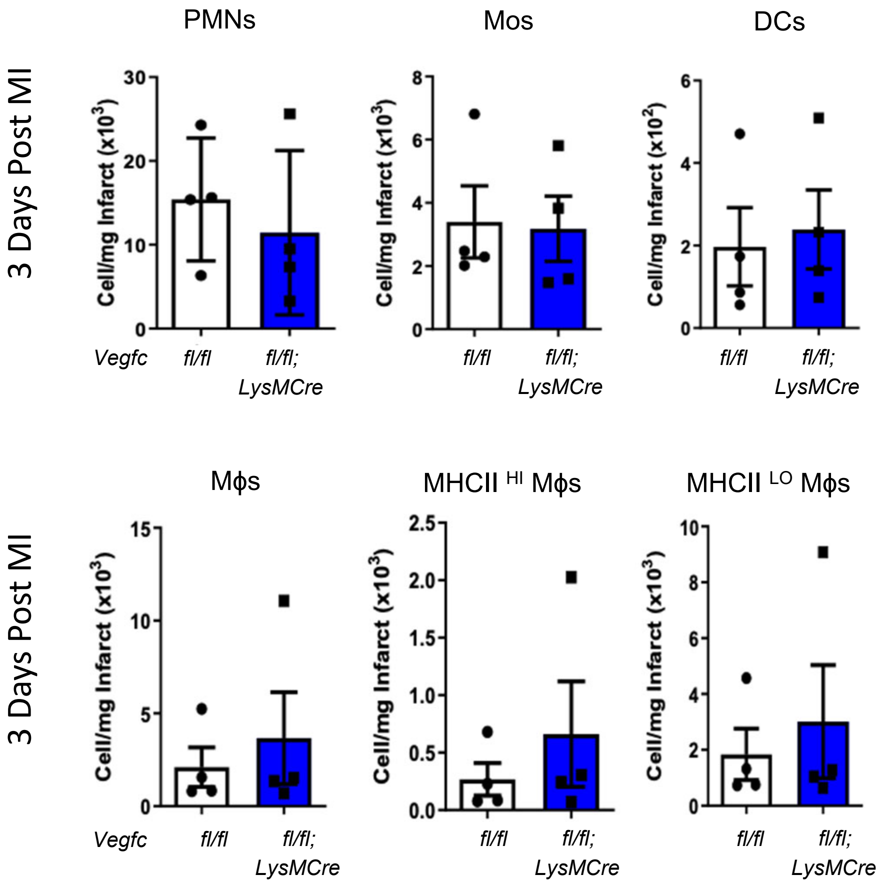


**Supplemental Figure 7. Day 3 levels of inflammatory cells post MI**. Flow cytometric analysis of indicated cardiac cell types at 3 days post-MI between *Vegfc*$^{fl/fl}$*;LysMCre* mice and *Vegfc*$^{fl/fl}$ littermate controls. n =4/group.

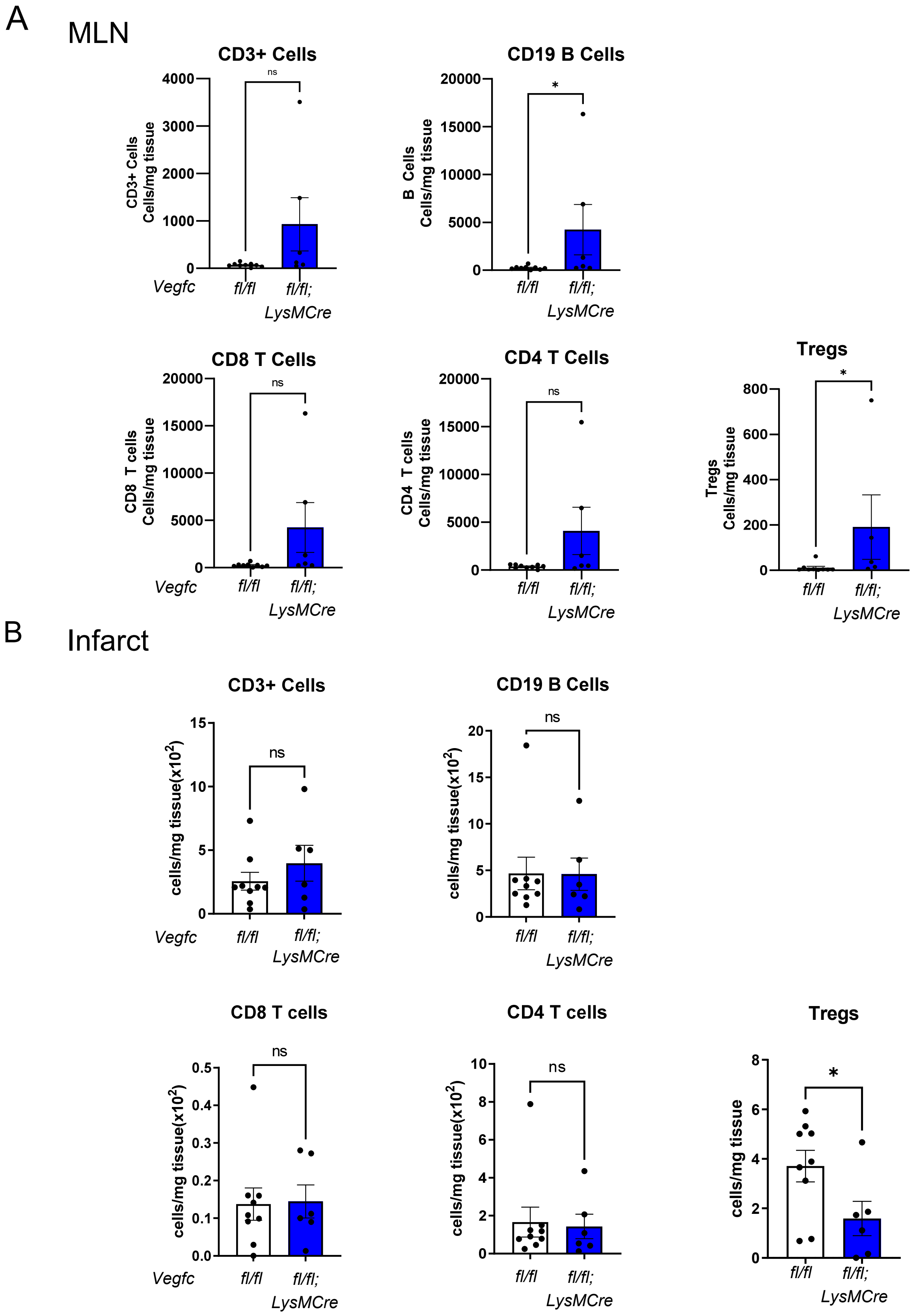


**Supplemental Figure 8. Myeloid VEGFC promotes infarct associated Tregs post MI**. (**A**) Flow cytometric analysis of lymphocytes in mediastinal lymph node (MLN) show higher level of B cells and Tregs in *$Vegfc^{fl/fl}$;LysMCre* mice compared to *$Vegfc^{fl/fl}$* littermate controls. (**B**) 7 days post-MI infarcts however had significantly lower Treg infiltration in *$Vegfc^{fl/fl}$;LysMCre* mice compared to littermate controls. n =6-8 per group.* $p < 0.05$ using Mann-Whitney U test.

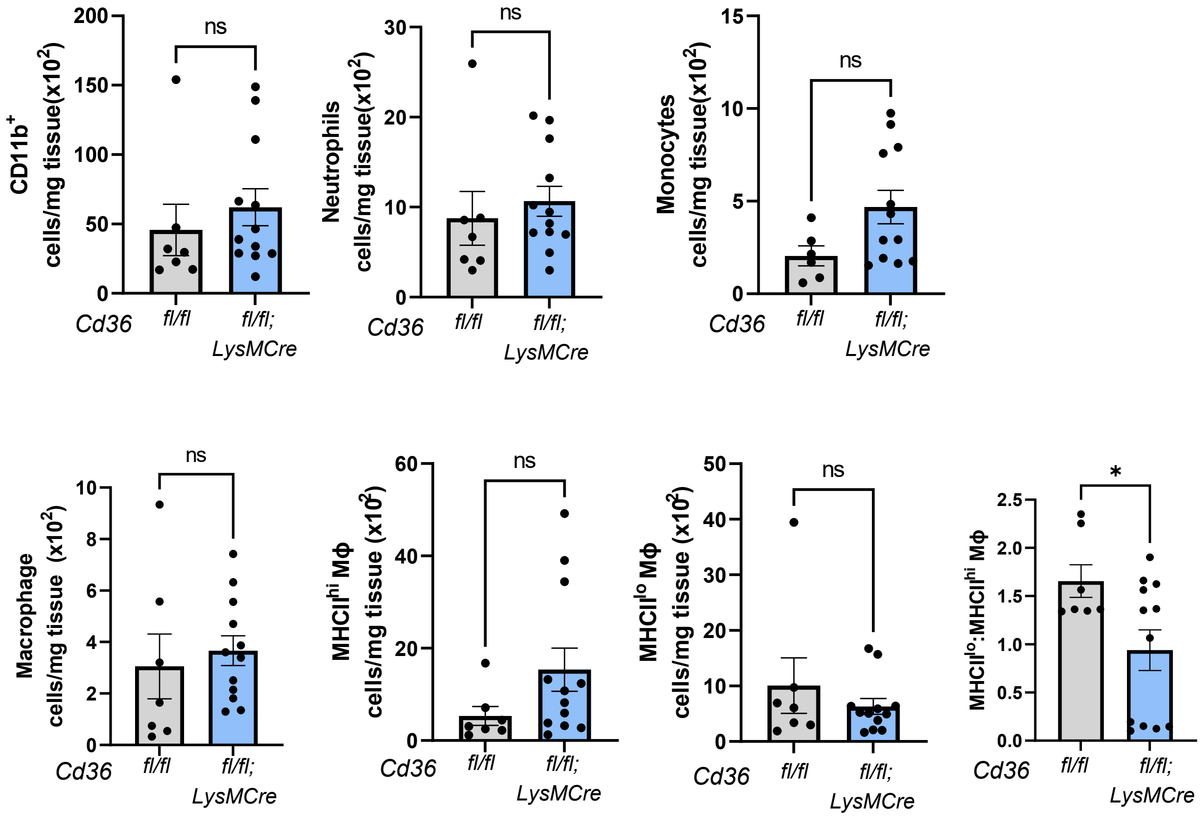


**Supplemental Figure 9. Evidence for heightened cardiac and macrophage inflammation in myeloid *Cd36*-deficient mice**. (**A**) Flow cytometric analysis of ischemic area at risk 7-days post MI in *Cd36* deficient mice revealed similar levels of CD11b$^{+}$Ly6g$^{+}$ neutrophils, Ly6c$^{hi}$ monocytes, and CD64$^{+}$F4/80$^{+}$ Mϕs compared to controls. Importantly, the ratio of MHCII$^{lo}$ to MHCII$^{hi}$ macrophages within the infarcted myocardium was significantly altered similarly to *Vegfc* deficient mice. * $p < 0.05$. n = 7-12 per group.

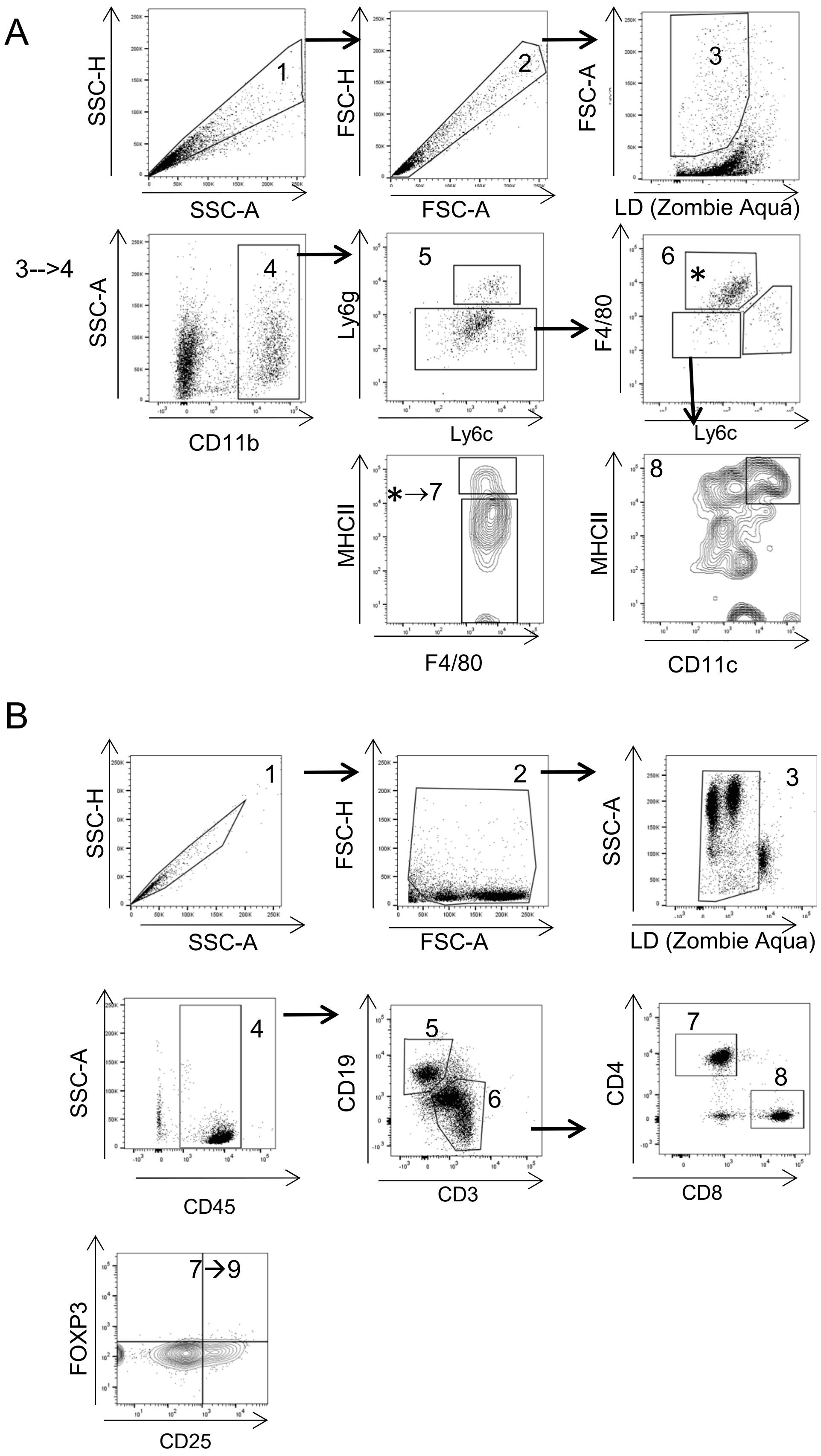


**Supplemental Figure 10. Gating strategy for myeloid and lymphoid cells in mouse hearts post myocardial infarction**. (**A**) Gating strategy for myeloid cells post coronary occlusion. After gating for doublets and dead cells, CD11b cells further separated by standard myeloid markers for levels of CD11b$^+$Ly6g$^+$ neutrophils, CD11b$^+$Ly6G$^{lo}$Ly6C$^{hi}$F4/80$^-$ monocytes, CD11b$^+$MHCII$^{hi}$CD11c$^{hi}$ DCs and CD64$^+$F4/80$^+$ Mϕs. (**B**) For lymphoid cells, CD45$^+$ cells were further separated using lymphoid markers. Cells were separated first by CD3 and CD19 expression (B cells). CD3 cells were further analyzed by CD4 and CD8 expression. Tregs were identified as CD4$^+$CD25$^+$FOXP3$^+$.

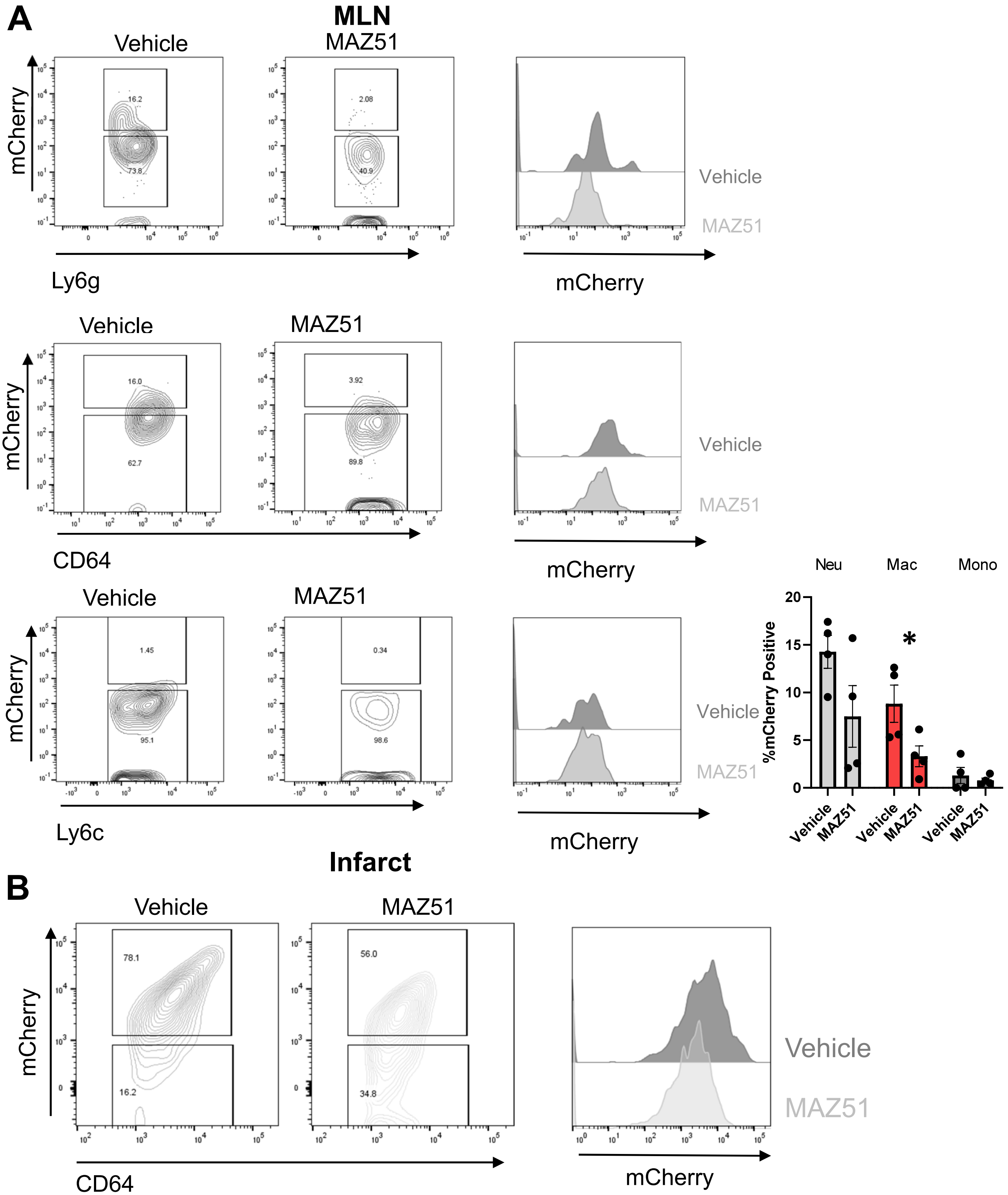


**Supplemental Figure 11. mCherry antigen detected in myeloid cells post myocardial infarction is affected by VEGFR3 inhibition.** (**A**) Post coronary occlusion flow cytometric analysis of medialstinal lymph nodes and relative levels of mCherry antigen uptake by Ly6g neutrophil-like cells, CD64 macrophages, and Ly6c monocyte-like cells, after treatment with VEGFR3 inhibitor MAZ51. (**B**) Flow cytometric analysis depcits decrease in mCherry antigen uptake by CD64$^{+}$ macrophages in infarct area after treatment with MAZ51 3 days post MI.

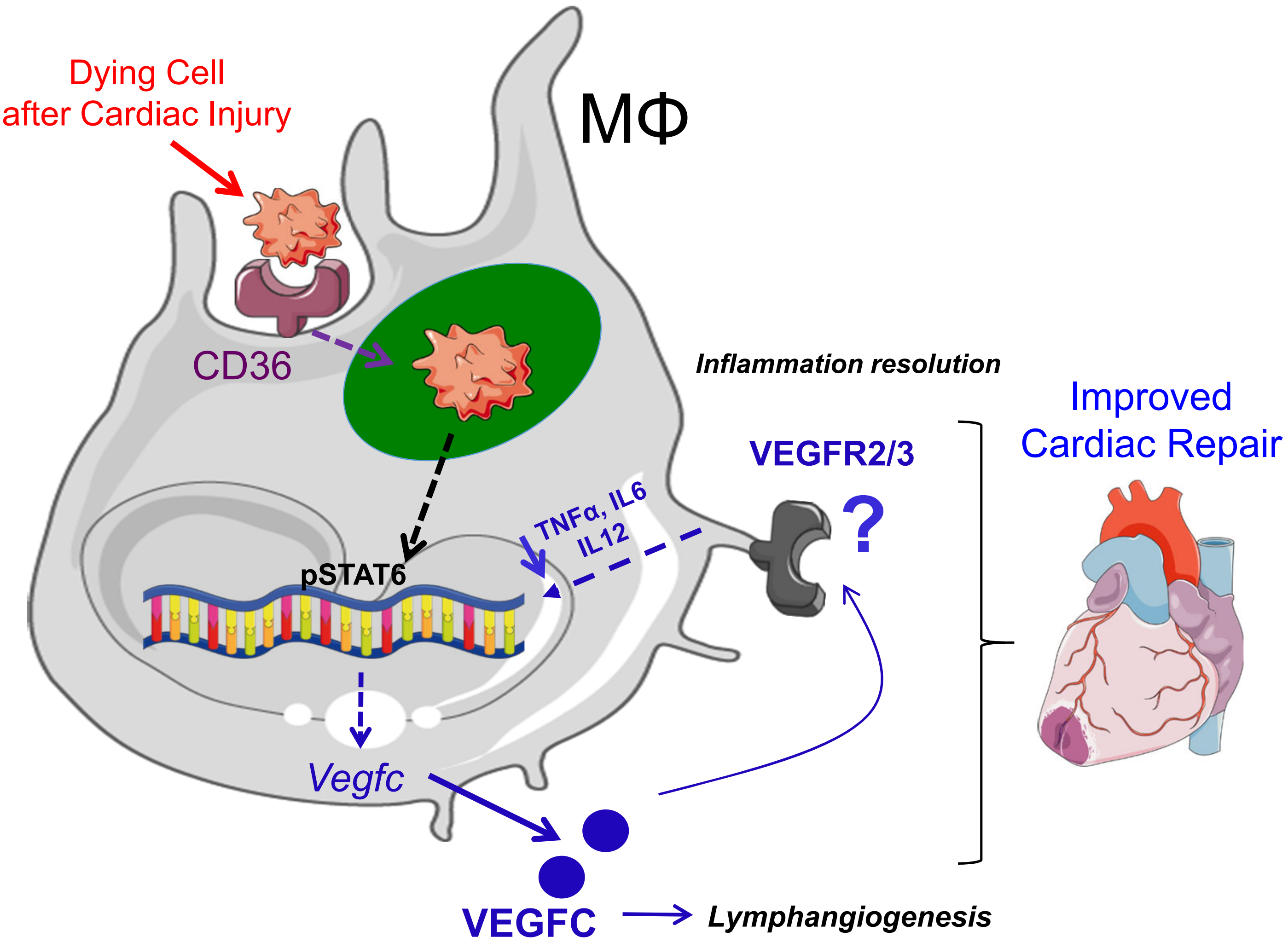


**Supplemental Figure 12. Working model based on experimental findings.** The schematic describes a scenario wherein cardiac damage after myocardial infarction, leads to phagocytosis of dying cells in the myocardium and the induction of macrophage VEGFC, leading to cardiac lymphangiogenesis and inflammation resolution. Macrophage = Mϕ.

Full uncut gels

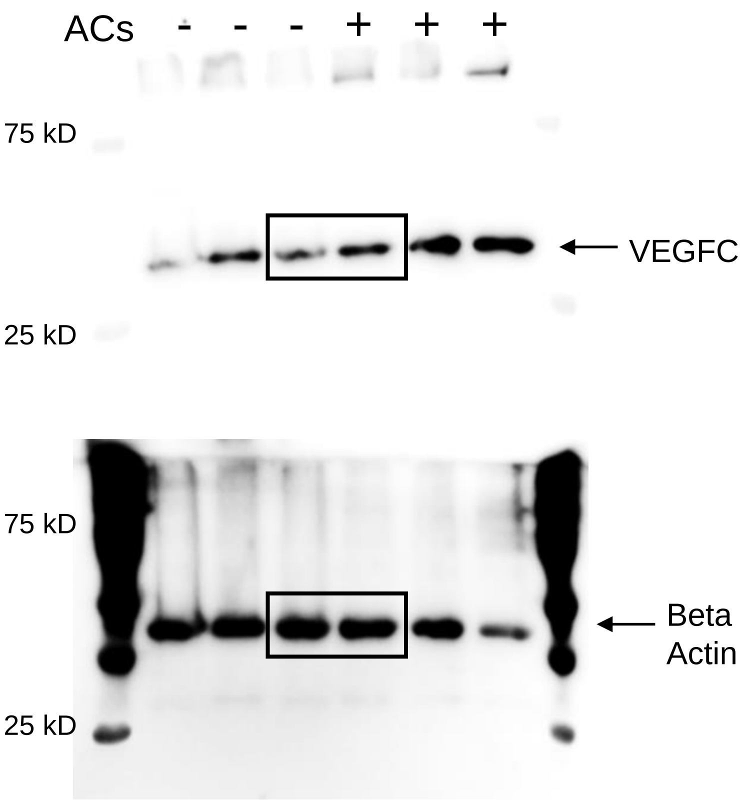


**Figure 2. *Vegfc* is induced in macrophages (Mϕs) during efferocytosis. (C)** Representative protein immunoblots of VEGFC 6 hours after efferocytosis and densitometry analysis.

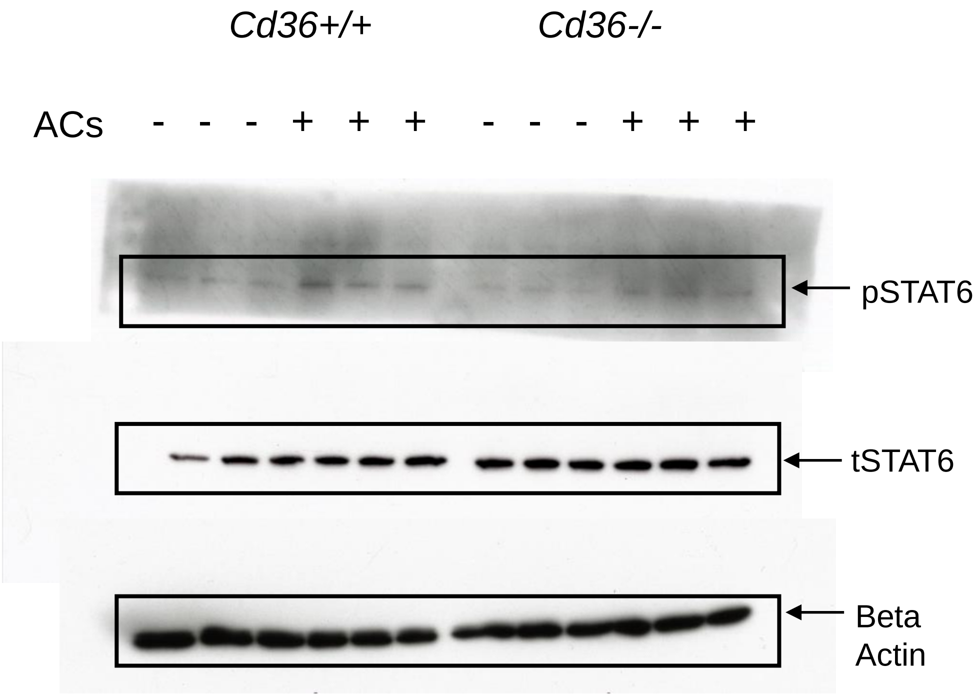


**Figure 2. *Vegfc* is induced in macrophages (Mϕs) during efferocytosis.** (**J**) To assess STAT6 phosphorylation Mϕs were cultured as above, and lysates were prepared in RIPA buffer then assessed by Western blotting.
